# Effectiveness and cost-effectiveness of a collaborative deprescribing intervention of proton-pump-inhibitors on community-dwelling older adults: Protocol for the C-SENIoR, a pragmatic non-randomized controlled trial

**DOI:** 10.1371/journal.pone.0298181

**Published:** 2024-03-26

**Authors:** Sónia Romano, Luis Monteiro, José Pedro Guerreiro, João Braga Simões, António Teixeira Rodrigues, Nuno Lunet, Julian Perelman

**Affiliations:** 1 Centre for Health Evaluation & Research/Infosaúde, National Association of Pharmacies (CEFAR-IF/ANF), Lisboa, Portugal; 2 NOVA National School of Public Health, NOVA University Lisbon, Lisbon, Portugal; 3 Centre for Health Technology and Services Research, Faculty of Medicine of the University of Porto (CINTESIS), Porto, Portugal; 4 Unidade de Saúde Familiar Esgueira Mais, Aveiro, Portugal; 5 Life and Health Sciences Research Institute (ICVS), School of Medicine, University of Minho, Braga, Portugal; 6 Unidade de Saúde Familiar Terra da Nóbrega, Ponte da Barca, Portugal; 7 ICVS/3B’s-PT Government Associate Laboratory, Braga/ Guimarães, Portugal; 8 Departamento de Ciências da Saúde Pública e Forenses e Educação Médica, Faculdade de Medicina, Universidade do Porto, Porto, Portugal; 9 EPIUnit—Instituto de Saúde Pública, Universidade do Porto, Porto, Portugal; 10 Laboratório para a Investigação Integrativa e Translacional em Saúde Populacional (ITR), Universidade do Porto, Porto, Portugal; 11 NOVA National School of Public Health, Comprehensive Health Research Center, CHRC, NOVA University Lisbon, Lisbon, Portugal; Jordan University of Science and Technology, JORDAN

## Abstract

**Introduction:**

Worldwide, demographic ageing is a major social, economic and health challenge. Despite the increase in life expectancy, elderly often live with multiple chronic conditions, exposing them to multiple medications. Concerns have been raised about the growing issue of inappropriate long-term usage of proton-pump inhibitors (PPI), which have been associated with adverse outcomes and increased healthcare costs. Deprescribing is a recommended intervention to reduce or withdraw medicines that might be causing harm or might no longer be of benefit. This protocol details a trial to assess the effectiveness and cost-effectiveness of a collaborative deprescribing intervention of PPI among community-dwelling elderly, involving community pharmacists and general practitioners.

**Methods and analysis:**

A pragmatic, multicentre, two-arm, non-randomised controlled trial of a structured PPI collaborative deprescribing intervention in the primary care setting with a 6-month follow-up will be conducted. Patients must be 65 years old or older, live in the community and have been using PPI for more than 8 weeks. We hypothesize that the intervention will reduce the PPI usage in the intervention group compared to the control group. The primary outcome is the successful discontinuation or dose decrease of any PPI, defined as a statistically significant absolute 20% reduction in medication use between the intervention and control groups at 3- and 6-month follow-ups. An economic evaluation will be conducted alongside the trial. This study was approved by the Ethics Research Committee of Nova Medical School, NOVA University of Lisbon and by the Ethics Committee from the Local Health Unit Alto Minho, Portugal.

**Discussion:**

This pragmatic trial will provide evidence on the effectiveness and cost-effectiveness of a patient-centred collaborative deprescribing intervention in the community setting in Portugal. It will also inform improvements for the development of future multi-faceted interventions that aim to optimise medication for the community-dwelling elderly.

**Clinical trial registration:**

ISRCTN 49637686.

## Introduction

Worldwide, demographic ageing is a major concern for social, economic, and health systems [1]. Despite an increase in life expectancy, older adults live with multiple chronic conditions, exposing them to numerous medications, thereby increasing the risk of medication non-adherence, drug-drug interactions (DDI), and use of potentially inappropriate medicines (PIM) [2–4].

PIMs are defined as a medication in which the risk of an adverse drug event (ADE) outweighs their clinical benefit, especially when safer or more effective alternatives are available [5]. Despite the development of guidelines and increased awareness among health professionals, approximately 25% of community-dwelling older European adults take at least one PIM [6], leading to an increased risk of ADEs, drug-related hospitalization, falls, and excess costs [7–11]. These findings highlight the need for improved interventions to reduce the prescription and use of PIMs.

Preventing harm due to medicines is currently a global patient safety challenge [12]. Deprescribing has gained attention as an approach to reduce polypharmacy and optimise medication use [13, 14]. It is an intervention supervised by health professionals aimed at reducing or discontinuing medications that may be causing harm or are no longer providing benefits [13, 15].

Medication deprescribing interventions have shown some positive outcomes [16], although several challenges arise during the process. Barriers are commonly reported such as uncertainty, adverse drug withdrawal events, and potential harm to the health professional-patient relationship.

However, community-dwelling older people appear to be willing to discontinue medications, particularly if assisted by their medical doctor and/or pharmacist, if taking multiple medications, if experiencing side effects, or believing that some medications are no longer needed [17–20]. Moreover, evidence suggests that better results are achieved when the deprescribing process includes a patient education component and involves cooperation among the prescriber, pharmacist, and patient [13, 15, 21, 22]. However, for the intervention to be scaled-up, the costs of providing the intervention to reduce PIMs must be offset by the added value for patients and the healthcare system.

Based on these findings, we developed a patient-centred deprescribing multidisciplinary approach focused on proton-pump inhibitors (PPI), a class of medications listed on several PIM lists for older people [23–26] and highly prevalent in the community-dwelling older people in Portugal [27]. We hypothesise that the intervention will reduce the use of PPI among intervention participants compared to the control group.

The C-SENIoR (Collaborative DepreScribing IntervENtion of PPI on community dwelling oldeR adults) trial will test whether a collaborative deprescribing intervention involving general practitioners (GPs) and community pharmacists (CPs) reduces the use of PPI among community-dwelling older adults and whether it is economically worthwhile, in a system marked by fragmentation across levels of care and providers. This paper details the protocol of the C-SENIoR trial.

### Objectives

The primary objective of the C-SENIoR trial is to assess whether a collaborative, multi-faceted deprescribing intervention results in superior PPI discontinuation or dose reduction at 3 and 6 months compared to usual care. Secondary objectives include evaluating the intervention’s cost-effectiveness; assessing its impact on overall medication burden (degree of polypharmacy and drug-drug interaction); analysing ADEs, adherence to therapeutics, and understanding participants’ knowledge and beliefs regarding PPI use; and documenting the time taken for PPI discontinuation compared to usual care. Participant satisfaction levels will also be assessed with the collaborative intervention and selected process outcomes (e.g., time elapsed until medical appointment), to evaluate the fidelity and quality of the intervention in the intervention group. Additionally, the trial seeks to explore how potential factors such as patients’ sociodemographic characteristics (e.g., sex, education level, self-rated health status, PPI treatment duration, etc.), baseline medication burden and beliefs about medicines may mitigate the intervention’s effectiveness.

## Methods

### Study design and setting

This is a pragmatic, multicentre, non-randomised 2-arm controlled trial with a 6-month follow-up. The intervention arm involves a collaborative deprescribing intervention, while the control arm follows usual care practices. The trial will be conducted in the community setting involving community pharmacies and Family Health Units (FHUs), in mainland Portugal. Community pharmacies are privately owned establishments staffed by licensed pharmacists. FHUs are primary care centres characterised by multidisciplinary teams with high clinical autonomy, whose performance is regularly evaluated through a wide range of indicators, and some FHUs professionals are partially compensated based on this performance.

To prevent contamination of the control group, a prospective geographic location-based method was employed. To minimize imbalance and improve causal inference, controls were matched with the intervention. Initially, areas closely matching the intervention settings based on patient characteristics were identified, using municipality sociodemographic characteristics as proxies (per capita Purchasing Power, Aging ratio, and Illiteracy rate) [28]. Subsequently, control FHUs in those areas, were identified by considering the FHUs’ contractual type with the National Health Service (NHS) and the prescription of PPI in Defined Daily Doses per 1000 inhabitants per day [29] for the registered elderly [30]. Finally, pharmacies surrounding these FHUs, with a client base from the eligible FHUs and using the dispensing software sifarma®, were considered. Due to the expected lower recruitment rate of pharmacies, the study will include more municipalities in the control arm to ensure a similar number of recruiting sites in both arms. A 1:1 ratio of participants (intervention: control) will be used.

**Fig 1:**
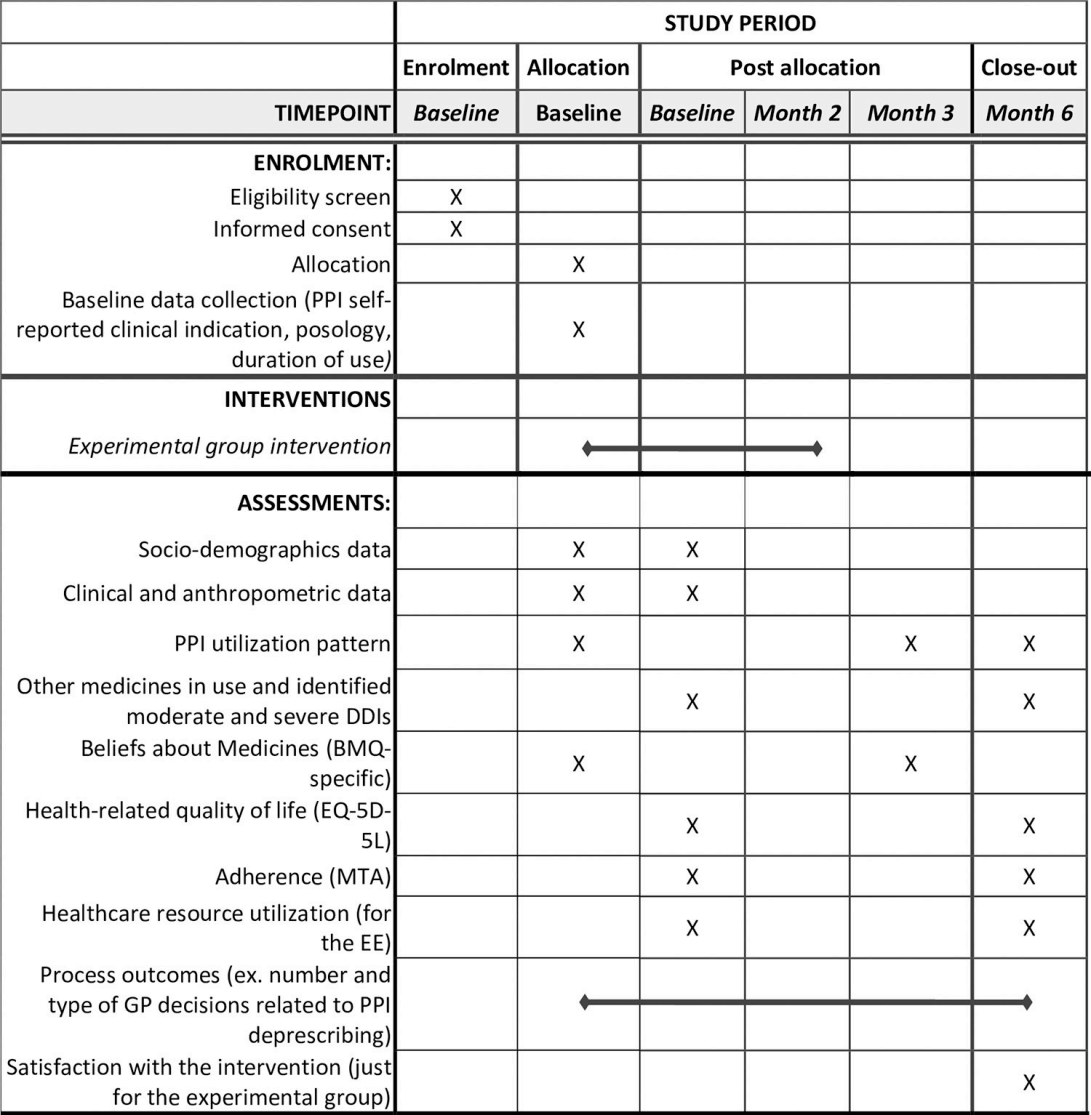
C-SENIoR study. SPIRIT 2013 schedule of enrolment, interventions, and assessments timepoints along the study period. BMQ-specific: Beliefs about Medicines Questionnaire; DDI: drug-drug interactions; EE: Economic evaluation; MTA: 7 items Measure Treatment Adherence questionnaire; EQ-5D-5L: 5-level EQ-5-dimension questionnaire.

**Fig 2:**
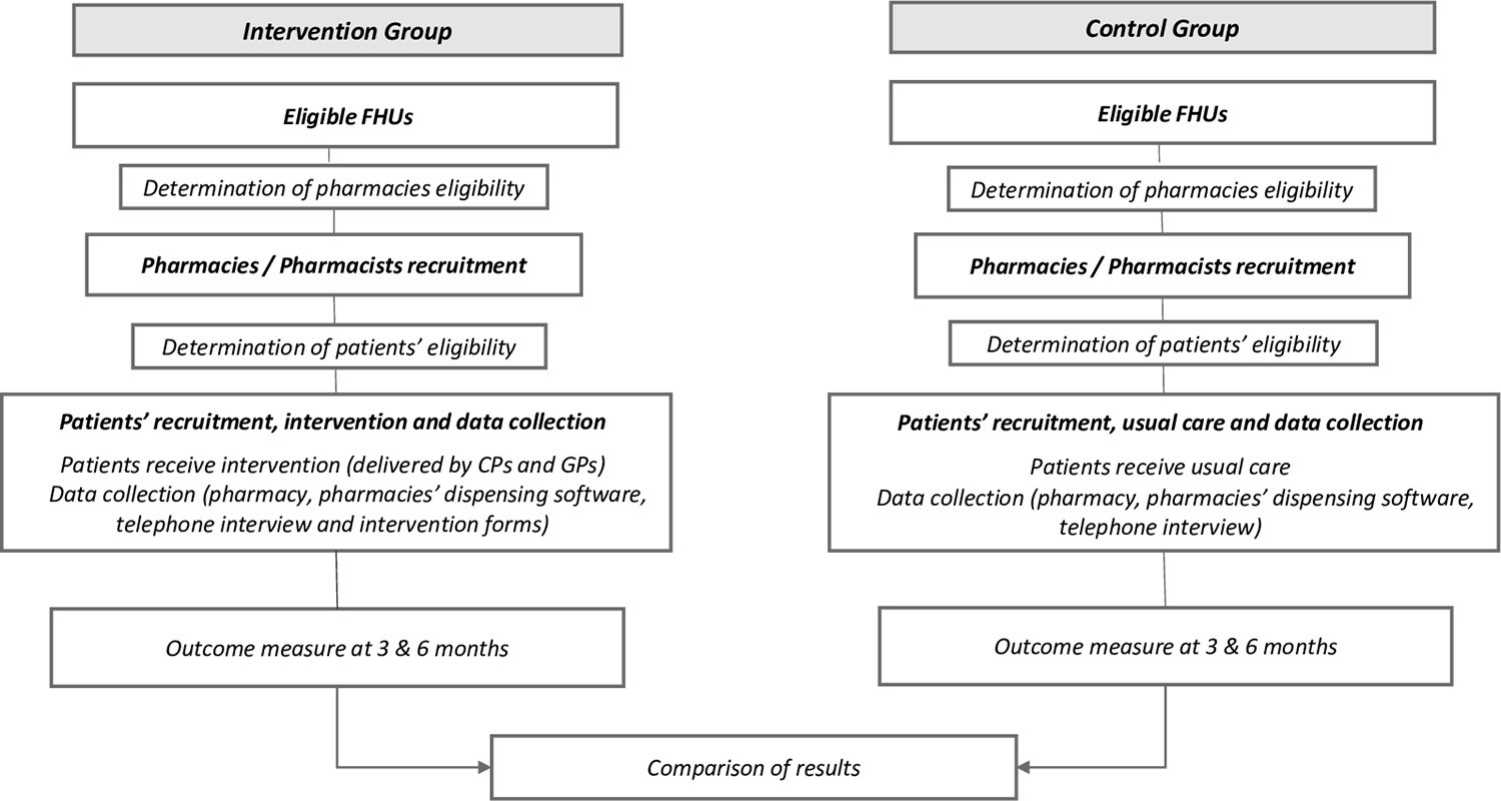
Flowchart of the C-SENIoR study. CPs: community pharmacists; FHUs: Family Health Units; GPs: general practitioners.

All eligible pharmacies will be invited to participate by email, followed by a telephone contact. The email will include a study information leaflet outlining the general study procedures. Figs 1 and 2 illustrate the C-SENIoR trial design.

An economic evaluation will be conducted alongside the trial, including the collection of cost data alongside the trial. The SPIRIT (Standard Protocol Items for Randomized Trials) reporting guidelines was followed on the drafting of the study protocol [31].

### Population

The eligible study population consists of community-dwelling older adults aged 65 years or older who are long-term users (>8-week use) of any PPI medication (ATC/WHO A02BC–esomeprazole, lansoprazole, omeprazole, pantoprazole, rabeprazole), are registered at the selected FHUs, and have access to a telephone. Participants will be recruited from community pharmacies in the municipality of the FHUs of interest, affiliated with the Portuguese National Association of Pharmacies (ANF), which represents approximately 95% of all pharmacies. The participant pharmacies must have a client base from the eligible FHUs and utilize the dispensing software sifarma®.

The PPI medication was selected based on: a) high consumption among the elderly in Portugal [27]; b) availability at lower doses as non-prescription medicine; c) preference by prescribers as a therapeutic group for starting to deprescribe [32]; d) alignment with international high-evidence criteria and recommendations for deprescribing [23, 24]; and e) existence of national clinical guidelines for reducing or discontinuing PPI use [33, 34].

Individuals who do not provide informed consent, reside in nursing homes or assisted-living facilities, are unable to communicate or speak in Portuguese, have cognitive impairments, or have any other condition that hinders their understanding of the study objectives or the questionnaire, will be excluded from the study.

#### Recruitment

In both intervention and control sites, the dispensing software (sifarma®) used by participating pharmacies will generate an electronic pop-up window whenever a PPI is dispensed. This will prompt the CPs to systematically assess the individuals’ eligibility criteria. If they meet the criteria, they will be invited to participate in the study and asked to sign an informed consent form. If an individual declines to participate, the pharmacist will complete a refusal form, recording basic sociodemographic information (sex and age group) and reasons for refusal.

To enhance the recruitment and adherence of pharmacies and patients, a comprehensive plan for follow-up contacts was established. This includes regular phone calls, emails, or in-person interactions with the pharmacies, aiming to remind the eligible criteria of the study population, provide reports on overall and individual recruitment statuses, offer feedback on data collection phone calls with enrolled participants, request assistance from pharmacies to engage with their recruited patients, and reinforce confidence in the study. During phone contacts with participants for data collection, emphasis will be placed on the importance of their contribution to knowledge generation, accompanied by expressions of appreciation.

### Intervention

**Fig 3:**
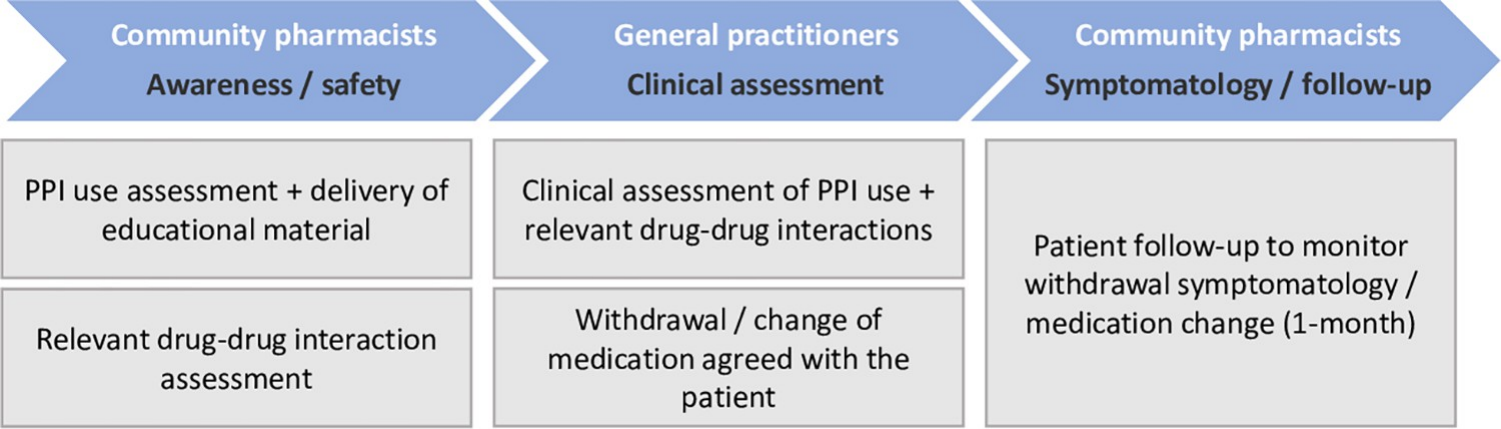
Overview of intervention in the C-SENIoR trial.

The intervention involves a collaborative care pathway between CPs and GPs to deprescribe inappropriate PPI and address other drug- related problems, such as DDIs. This patient-centred intervention package comprises three main components: the first is aimed at educating patients, raising awareness, and ensuring safety; the second is focused on making the clinical assessment and decision to deprescribe; and the third is designed to ensure patients’ follow-up during the withdrawal process (Fig 3).

1. Education, awareness, and safety

First, the CPs will assess the potential inappropriate use of PPI based on the patient’s self-reported clinical indication for its use and the deprescription algorithm developed by the research team. Second, the CPs will provide in-person oral and written educational information directly to the patient using a booklet. This booklet covers topics such as the rational use of medicines, indications and possible harms of chronic PPI use, benefits of deprescribing, potential withdrawal symptoms, and strategies to minimize these symptoms. The CPs and the GPs in the research team specifically developed the booklet for this study. To ensure its quality, the literature was reviewed of resources that have already been tested in deprescribing interventions and available online (e.g., https://deprescribing.org/). We adhered to the recommendations of Fajardo et al. [35] for the readability of patient education materials in deprescribing interventions during the development process. The booklet was pre-tested with six older long-term PPI users in collaboration with two pharmacies not involved in the trial. The pre-testing aimed to evaluate the booklet’s readability, dimensions, comprehension of key messages, and understanding of images.

Third, following patient recruitment and educational intervention at the pharmacy, the research team will generate a semi-automatic therapeutic profile of each patient’s chronic medications, which include relevant information about DDIs. This data will be extracted from the pharmacies’ dispensing software (sifarma®), using the patients’ tax information number. The therapeutic profile will be validated with the participants through a telephone interview conducted by the research team with the participant. The final therapeutic profile and related safety information will be sent via email to the CPs to be further shared with the patient’s GP for clinical assessment.

Fourth, the CP will compile all the collected information, including the PPI name, indication of use, evidence-based deprescribing recommendation, and the therapeutic profile with identified moderate and severe DDIs. The CP will forward this information to the patient’s GP using a specific paper case report form called the "Patient’s Passport". As the software used by the CPs and GPs do not communicate directly, this paper-based exchange of information ensures effective communication between providers.

2.Clinical assessment / deprescribing

Fifth, using the information provided by the CPs and accessible from the patients’ medical records, the GP will evaluate the usage of PPI and address any reported safety concerns. Subsequently, the GP will contact the patient, preferably by telephone, to discuss the appropriateness and strategy for deprescribing PPI following the national guidelines, as well as to address any other medication-related issues. If necessary, the GP may schedule a face-to-face appointment. Sixth, the GP will communicate all relevant information regarding medication safety, the decision to withdraw PPI and the agreed-upon strategy to the CPs using the Patient’s Passport.

3. Monitoring (symptoms/ follow-up)

Seventh, the CPs will conduct telephone follow-ups with the patients at 2 and 4 weeks after their appointment with the GP. These follow-ups aim to assess possible symptoms relapses, address patient inquiries, and establish pharmacological and non-pharmacological symptom management strategies (e.g., reviewing dietary intake) outlined in the intervention procedures. The 4-week telephone interview will be conducted only for patients recommended a reduction or withdrawal of PPI dosage.

Eighth, at the end of the withdrawal follow-up period, the CPs will share the patient’s monitoring information with the GP using the Patient’s Passport. As this is a pragmatic trial, some flexibility is allowed. Patients can seek advice from the CP and/or the GP at any time. Additionally, if any severe symptoms are identified during the intervention period, the patient will receive assistance from the GP. Anticipated low probabilities of symptom relapses or complications associated with medication withdrawal have been considered [36, 37]. Printed materials on the intervention flowchart, deprescribing guidelines, and procedures will be provided to professionals in the intervention arm to follow.

The project will be supported by a Clinical Research Associate (CRA) who will facilitate the exchange of information between pharmacies and the FHUs in the intervention arm. The CRA will also monitor and assist in clarifying recruitment and intervention procedures across centres to ensure protocol consistency.

The comparator in this study is usual care. In both study arms, a baseline paper-based questionnaire will be provided by the CPs during recruitment, and a telephone interview will be conducted by the research team within the first week after enrolment.

### Procedures

#### Blinding

Due to the nature of the intervention, blinding of the CPs, GPs, and patients is not feasible. However, the control group is blinded by design. The research team responsible for the development and monitoring of the trial will neither be blinded. Nevertheless, to prevent bias in the evaluation of the outcome measures, the statistician involved in the study will conduct a blinded analysis to ensure an objective evaluation of the study’s outcomes.

#### Training

Prior to the trial initiation, the GPs and CPs in the intervention group will undergo a mandatory, in-person training session regarding the study and intervention procedures, delivered by the researchers. In the control group, CPs will receive a briefer training focused solely on the study procedures, including patient eligibility criteria, enrolment strategy, and application of the baseline questionnaire. All study materials will be delivered to participant pharmacies by hand or via express mail before the training sessions.

#### Data collection

A baseline paper-based questionnaire will be conducted at the pharmacies after patient recruitment and before the intervention. Additionally, a baseline telephone questionnaire will be conducted within the first week after enrolment. Two additional telephone follow-ups will be conducted at 3 and 6 months. Electronic data will be extracted from pharmacies’ dispensing software at baseline and 6 months post-recruitment. Trained research assistants, blinded to group allocation, will conduct telephone interviews expected to last between 15 and 20 minutes. Data will be digitally recorded and securely stored for analysis. For process outcomes analysis and economic evaluation, data will also be collected from the “Patient’s Passport” and pharmacists’ case reports.

#### Data management

Data security measures will be implemented to ensure the confidentiality and integrity of collected information. Paper-based questionnaires and other physical data will be securely stored in a locked cabinet and subsequently transferred to a secure electronic database with restricted access for researchers. Telephone questionnaires data will be collected digitally and stored in the electronic database. Patients will be requested to grant permission for medication data extraction from the pharmacies dispensing software. Before analysis, data sources will be anonymized in accordance with the General Data Protection Regulation (GDPR) guidelines on personal data handling. A Data Privacy Impact Assessment of this study was conducted by the researchers and reviewed by the Data Protection Officers of Infosaúde/ANF. Data protection procedure agreements were signed by both Infosaúde/ANF and Local Health Unit Alto Minho, the entity responsible for the FHUs. All members of the research group undertook training in GDPR and trial procedures.

### Outcomes and variables

The outcomes are measured at patient level. The primary outcome is the successful discontinuation or dose decrease of any PPI, defined as a statistically significant absolute 20% reduction in medication use between the intervention and control groups at 3- and 6-months follow-ups, ascertained by the pharmacies medicine sales associated to the patients’ tax information number and confirmed by patient telephone interviews.

Secondary outcomes are:

- Time until complete withdrawal since recruitment [38].

Assessment at baseline and 3-month follow-up:

- Patients’ beliefs about inappropriate medicines regarding PPI, measured by the Beliefs about Medicines Questionnaire (BMQ-specific) [39].

Assessment at baseline and 6-month follow-up:

- Number of long-term medications and proportion of patients on polypharmacy (patients with 5 or more medicines [40]).
- Health-related quality of life (HRQoL), based on EQ-5D-5L instrument [41, 42].
- Self-reported adherence measured by the 7 items Measure Treatment Adherence questionnaire (MTA) [43].
- Number and degree of severity of DDIs identified by the dispensing software.
- ADEs, including the absolute and relative counts of self-reported ADEs and type of events (e.g., requiring professional support) experienced by patients.
- Satisfaction with the collaborative intervention (both general and health professional related support) for the intervention group, assessed by a 5-level Likert scale ranging from”1- Not at all satisfied” to “5-Very satisfied”, only at the 6-month follow-up.

Process outcomes, such as the number and type of GP decisions related to PPI deprescribing and the number of pharmacist telephone follow-ups, will be collected ongoing to assess the fidelity and quality of the collaborative intervention.

### Sample size

The sample size calculation was based on the hypothesis that the intervention will result in a proportion of patients with PPI discontinuation that is at least as high as that achieved in previous withdrawal studies involving collaborative efforts between CPs and GPs, compared to usual care. Specifically, we aim to detect a minimum absolute difference of 20% in the proportion of patients undergoing PPI discontinuation or decreased dosage at the 6-month follow-up [22, 44].

Considering that approximately 10% of the users who do not receive the intervention may naturally discontinue PPI, with an alpha of 0.05 and 90% power, and assuming an allocation ratio of 1:1, we estimated that the minimum total sample size to detect differences between intervention and control groups is 178 patients (89 patients per group). Accounting for potential losses to follow-up of 20% of the patients [45], a total of 222 participants (111 per group) will be needed.

### Statistical analysis

A statistical analysis plan will be drafted before any analysis takes place. The null hypothesis proposes no difference in primary outcomes between intervention and control patients. An intention-to-treat approach will be considered, including in the analysis patients regardless of the degree to which they have been exposed to the intervention (as this is a pragmatic trial). Outcomes will be estimated for the whole dataset. 95% confidence intervals will be reported.

The description of the baseline characteristics (sex, education level, self-rated health status, treatment duration, etc.), and primary and secondary endpoints will be presented for all patients and stratified by arm and other subgroups (based on the categorisation of beliefs about medicines, medication burden). Comparisons between arms will be performed using the chi-square/Fisher test for categorical variables and/or t-test/ANOVA or nonparametric Wilcoxon/Kruskal-Wallis test for continuous variables. These will be used to assess the balance between the groups on these possible study confounders.

The primary outcome of the study will be calculated using a GLM model for binary outcome with an identity link function to estimate the risk difference or the difference between intervention and control groups in the proportion of patients who discontinued or decreased PPI dosage at 6-month follow up. To account for potential confounding, the results will be adjusted for baseline covariates. Relative risk will be calculated as well as the number needed to treat (NNT)–the inverse of the difference in absolute rate of discontinuation between the intervention and control groups. Analysis will also be conducted at 3-month follow up.

Regarding secondary outcomes, descriptive statistics will be calculated for all patients and reported with respect to each time point. For each outcome, adequate GLM models will be used to compare groups with respect to therapeutic outcomes (PPI specific and other medication), utilities derived from the EQ-5D-5L, beliefs about PPI (BMQ specific), adherence (MAT), and healthcare resource utilization. Changes over time points will be evaluated.

Time to PPI discontinuation will be accessed through Kaplan-Meier (KM) estimator. Results will be stratified by group and KM curves will be presented. Log-rank test will be computed to compare results between cohort subgroups. In alternative, multivariate Cox Proportional Hazards models will be used if groups are unbalanced and hazard ratios computed.

The analysis of participants’ satisfaction level and process outcomes will be limited to the intervention arm of the study. These data will be used to assess compliance with the intervention design and the participants’ acceptance of this intervention type, aiming to enhance the design of future collaborative models.

To further assess internal validity, participants will be compared with those who refused to participate, taking into consideration sex and perceptible age class.

Statistical analysis will be performed in SAS Enterprise Guide 7.15 (Cary, NC) and R Software. The significance level adopted is α = 0.05.

### Cost-effectiveness study

A trial-based economic evaluation (EE) will be performed to assess the cost-effectiveness of C-SENIoR compared to current practice. In the base-case scenario, the EE will adopt a NHS perspective, considering the direct costs of providing the intervention, healthcare resource use (HCRU) costs and medication expenses in accordance with Portuguese guidelines [46]. A broader perspective, including out-of-pocket expenses, will be explored in a scenario analysis. The base-case analysis will consider a time horizon of 6 months.

The outcomes will be based on the primary effectiveness outcome measure—the proportion of patients who discontinued PPI or decreased PPI dosage in the 6-month follow-up -, and on the EQ-5D-5L. This instrument will be used to derive summary index values based on the Portuguese value set and calculate Quality-adjusted life years (QALYs) for a cost-effectiveness analysis with results expressed as euros per QALY [42]. The 5-level EQ-5D version of the generic preference-based measure of health state was chosen because it has demonstrated improved reliability, sensitivity (discriminatory power), and feasibility compared to the previous three-level instrument [47].

The cost of the intervention, such as training of CPs and FHU staff, number and type of GP’s appointments, time spent by the CPs preparing and delivering the intervention, undertaking administrative or other miscellaneous tasks, will be estimated considering data recorded by the pharmacists and GP’s in the intervention forms filled at each step of the intervention, valued through established methods [46, 48] using official tariffs [49, 50]. Information on HCRU will be collected at the patient level through the pharmacies’ dispensing software and telephone questionnaires administered to patients at baseline and 6-month follow-up. At baseline, HRCU will encompass the preceding 6 months before recruitment, and the 6-month follow-up will cover the period since the baseline measurement. HCRU include medication, primary and secondary healthcare provider visits, medical exams, emergency visits, hospitalization, and surgery. Information on prices of medicines will be collected from the pharmacies’ dispensing software, while all other items will be valued using official NHS prices [50]. All costs will be reported in Euros (€).

An incremental cost-effectiveness ratio (ICER) will be calculated by dividing the incremental costs by the proportion of inappropriate PPI that has been discontinued or decreased dosage in the cost-effectiveness analysis and by the QALYs gained. Deterministic sensitivity and scenario analyses will be conducted for all parameters with uncertainty; a probabilistic sensitivity analysis will also be performed, and a cost-effectiveness acceptability curve designed [51].

The cost-effectiveness of the intervention will be defined based on existing public thresholds, such as the ones applied by the UK NICE or by the WHO, in the absence of a public threshold in Portugal [52].

The Consolidated Health Economic Evaluation Reporting Standards (CHEERS) will be followed for reporting the EE [53].

### Ethics

This study was approved by the Ethics Research Committee of Nova Medical School, Faculty of Medical Sciences, NOVA University of Lisbon (16/2021/CEFCM) and by the Ethics Committee for Health from the Local Health Unit Alto Minho (50/2022/CES), Portugal.

A written informed consent will be obtained from all participants, in accordance with local practice and regulations. All participation is voluntary, and subjects can withdraw fully or partially from the study at any time and for any reason, without jeopardizing the delivery of patient care. The trial will be conducted in compliance with the ethical principles mandated by the 2008 Declaration of Helsinki and its subsequent modifications, as well as Good Clinical Practice guidance. Any amendment or deviation from the protocol will be reported to the Human Research Ethics Committee. No payments were made or will be made to researchers, GPs, CPs, or patients for their involvement in this trial.

The protocol was retrospectively registered at ISRCTN registry (ISRCTN49637686). This occurred due to multiple study start date postponements, site relocation, and a lack of awareness about the need of prior registration. Registration occurred shortly after commencing recruitment. The authors confirm that all ongoing and related trials for this drug/intervention are registered.

### Dissemination and impact

The project is planned to include the publication of at least three scientific articles in peer-reviewed journals and presentations at both national and international conferences. Additionally, we plan to engage with key stakeholders including local and national health policymakers (Directorate-General for Health, National Authority of Medicines and Health Products, National Health Service Executive Board, Central Administration of the Health System), to ensure the dissemination of the study findings and advocate for the development of collaborative interventions to improve medication use. Furthermore, we intend to provide feedback to all the participants using established communication channels and the public through the media.

### Current trial status

Recruitment of participants began on 28^th^ April 2023, and was approximately 60% complete at the time of this protocol submission. The patient recruitment period extended until 15^th^ November 2023, with data collection expected to run until June 2024.

## Discussion

This pragmatic trial involves the collaboration between public FHUs and private community pharmacies, a condition not typically observed in the highly fragmented Portuguese health system. We anticipate that this intervention will optimise therapeutics for older individuals. Overall, the study will generate evidence on the effectiveness and cost-effectiveness of deprescribing interventions in community setting. Additionally, it will provide insights for enhancing future collaborative interventions designed to optimise medication use in the elderly within a real-world clinical practice setting.

The collaborative efforts of health professionals, emphasizing a patient-centred approach, aim to reinforce patients’ decision-making and endorse the intervention by providing comprehensive knowledge about the risks, benefits, and potential discontinuation of their medicines.

This trial also has some limitations. An experimental design with random assignment was not feasible because the intervention model was developed based on the expressed interest of two FHUs to integrate a collaborative project with pharmacies. These FHUs are located in distinct small municipalities, with few primary healthcare settings. Additionally, the scarcity of surrounding pharmacies made randomization impractical. Finally, randomization at the patient level was not feasible due to the high risk of contamination of the control group. To address these limitations, we implemented measures such as geographical separation and matching of controls that will also prevent contamination between the experimental and control groups. Additionally, to account for potential confounding several covariates will be collected to ensure adequate control.

Furthermore, the study invitation, focused on long-term PPI use rather than a specific diagnosis, poses a risk of recruiting a considerable number of patients with a legitimate indication for PPI use, who may not meet deprescribing criteria.

Selection bias may also occur based on the perceived benefit or harm of participating in the study. The stringent inclusion criteria and the advanced age of the study population pose challenges in recruiting and retaining participants, as these are influenced by various factors, such as communication issues, distrust of research studies, health-related concerns, dissatisfaction with data collection procedures, a lack of incentives for control group participants, and apprehension about potential adverse events among those undergoing the intervention [54]. To minimize this effect, a comprehensive plan for follow-up contacts was implemented.

Moreover, there is a risk of recall bias in this trial due to the use of self-reported questionnaires, requiring participants to recall and report retrospective information on their previous HCRU. Despite this risk, the self-report approach is necessary given the nature of the data being collected.

Finally, it is important to acknowledge that this study is limited in generalizability to other medications, settings, and populations since it will take place in a specific region of Portugal.

## Supporting information

S1 File SPIRIT 2013 checklist. Recommended items to address in a clinical trial protocol and related documents.
(DOC)

S2 File Project submitted to the ethics committee (originally submitted in English). 22 July 2022. Version 01.1.
(PDF)
