# Supplementary material for: Effectiveness and cost-effectiveness of a collaborative deprescribing intervention of proton-pump-inhibitors on community-dwelling older adults: Protocol for the C-SENIoR, a pragmatic non-randomized controlled trial
Source: PLoS One. 2024 Mar 26;19(3):e0298181. doi: 10.1371/journal.pone.0298181 (PMC10965080; doi:10.1371/journal.pone.0298181)
Supplement: S2 File — 22 July 2022. Version 01.1. (PDF) [file pone.0298181.s002.pdf]

Centro de Estudos e Avaliação em Saúde

Escola Nacional de Saúde Pública
Universidade Nova de Lisboa

Interventional Study Protocol

TITLE: Evaluating the effectiveness and cost-effectiveness of a Collaborative depreScribing intervENtion of PPI on community dwelling eldeRly: a study protocol for the C-SENIoR, a pragmatic non-randomized controlled trial

Funding:	Portuguese National Association of Pharmacies (ANF) / Infosaúde
Sponsor:	ANF
Trial registration	Intended registry - ISRCTN registry
Version number:	v01.1 (revised protocol)
Date:	22 July 2022

Authors | Affiliation

Sónia Romano | Centro de Estudos e Avaliação em Saúde (CEFAR/Infosaúde), Associação Nacional das Farmácias (ANF), Lisboa, Portugal; Escola Nacional de Saúde Pública, Universidade Nova de Lisboa (ENSP/UNL), Lisboa, Portugal.

Luis Monteiro | Centre for Health Technology and Services Research / Faculty of Medicine of the University of Porto (CINTESIS), Porto, Portugal; Unidade de Saúde Familiar Esgueira Mais, Aveiro, Portugal.

José Pedro Guerreiro | Centro de Estudos e Avaliação em Saúde (CEFAR/Infosaúde), Associação Nacional das Farmácias (ANF), Lisboa, Portugal

João Braga Simões | Life and Health Sciences Research Institute (ICVS), School of Medicine, University of Minho, Braga, Portugal; Unidade de Saúde Familiar Terra da Nóbrega, Ponte da Barca, Portugal.

António Teixeira Rodrigues | Life and Health Sciences Research Institute (ICVS), School of Medicine, University of Minho, Braga, Portugal; ICVS/3B's-PT Government Associate Laboratory, Braga/ Guimarães, Portugal.

Nuno Lunet | EPIUnit - Instituto de Saúde Pública, Universidade do Porto, Porto, Portugal. Departamento de Ciências da Saúde Pública e Forenses e Educação Médica, Faculdade de Medicina, Universidade do Porto, Porto, Portugal.

Julian Perelman | Escola Nacional de Saúde Pública, Universidade Nova de Lisboa (ENSP/UNL), Lisboa, Portugal.

Table of Contents

1. Protocol Summary 6
2. Amendments and updates 12
3. Background and Rationale 14
4. Aim and objectives 18
Primary objective 18
Secondary objectives 18
Exploratory objectives 18
5. Study Design 19
6. Methods 20
PARTICIPANTS, INTERVENTIONS, AND OUTCOMES 20
6.1. Study setting 20
6.2. Pharmacy Invitation / Recruitment 23
6.2.1. Retention Strategies 23
6.3. Study population and Eligibility criteria 23
6.3.1. Individuals' inclusion Criteria 24
6.3.2. Exclusion Criteria 24
6.4. Study Enrollment and Patient Recruitment Procedures 24
6.5. Intervention and control 25
6.6. Participant timeline 28
6.7. Study Follow-up 29
6.8. Outcomes 29
6.9. Blinding 32
6.10. Potential harms and patient safety 33
6.11. Sample size 33
DATA COLLECTION, MANAGEMENT, AND ANALYSIS 37
6.12. Data collection, Variables and Definitions 37
6.13. Data management and Quality control 45
6.14. Data recording and document retention 46
6.15. Statistical Methods 46
6.15.1. General principles 46
6.15.2. Study outcomes 47
Primary outcomes 47
Secondary outcomes 47
6.15.3. Enrolled population and refusing subjects. 48

6.15.4. Population of analysis 48
6.15.5. Timing of analysis 48
6.15.6. Statistical software 48
6.16. Site monitoring 49
6.17. Economic evaluation 50
6.17.1. Overview 50
6.17.2. Measurement and valuation of resources 50
6.17.3. Intervention costs 51
6.17.4. Outcomes for the economic evaluation 51
6.17.5. Cost and cost-effectiveness analysis 52
7. Study limitations 53
8. Safety Reporting and Related Procedures 55
8.1. Adverse Events and Serious Adverse Events 55
8.2. Adverse Event Reporting 55
9. Ethics and dissemination 55
9.1. Research ethics approval 56
9.2. Informed consent procedures 57
9.3. Confidentiality of study/subject Data 58
9.4. Declaration of interests 58
9.5. Funding 59
9.6. Disseminating Policy 59
References 60
Annexes 67
Supplemental study materials – Stand-alone documents 67

List of Tables

Table 1 – Study protocol amendments and updates regarding previous approves by CEFCM-UNL.... 12
Table 2 - Planned dates of study milestones 13
Table 3 - Details of the intervention and identified municipalities and control FHUs........................ 22
Table 4 - Overview of data collection schedule and measurements 32
Table 5 – List of variables 37
Table 6 - List of stand-alone documents (Annexes) 67

List of Figures

Figure 1 - Study Flowchart 19
Figure 2 - Overview of the C-SENIoR multifaceted collaborative intervention package 27
Figure 3 - Average number of PPI sales per pharmacy in intervention Municipalities Jan 21-mai 22 (hmR sell-out data).. 35

List of abbreviations

ACES	Agrupamento de Centros de Saúde
ADE	Adverse Drug Event
ADWE	Adverse drug withdrawal event
ANF	Portuguese Association of Pharmacies
AQL	Acceptance Quality Level
ATC	Anatomical Therapeutic Chemical classification
BMQ-Specific	Beliefs about Medicines Questionnaire
BMI	Body mass index
CEFAR	Centre for Health Evaluation & Research
CRA	Clinical Research Associate
C-SENIoR	Collaborative Deprescribing Intervention of PPI on community dwelling elderly
DDI	Drug-Drug interactions
EC	Ethics Committee
EQ-5D-5L	Euro-Qol five-dimension five-level
EU	European Union
GERD	Gastroesophageal reflux disease
GP	General Practitioner
HRQoL	Health-Related Quality of life
ICF	Informed Consent Form
ICU	Intensive Care Unit
LOS	Length-of-stay
MAT	7 items Measure Treatment Adherence
NHS	National Health Service
NPM	Non-prescription medicines
OOPE	Out-of-pocket expenditures
OTC	Over the counter (non-prescription medicines)
PIA	Privacy impact assessment
PIM	Potentially inappropriate medication
PIP	Potentially inappropriate prescription
POM	Prescription only-medicines
PPI	Proton-pump inhibitor
PREM	Patient reported experience measures
PROM	Patient-Reported Outcome measures
GDPR	General Data Protection Regulation
SAP	Statistical Analysis Plan
SD	Standard deviation
SRH	Self-rated health status
ULSAM	Unidade Local de Saúde do Alto Minho
FHU	Family practice units
WHO	World Health Organization

1. Protocol Summary

Title	Evaluating the effectiveness and cost-effectiveness of a Collaborative depreScribing intervENtion of PPI on community dwelling eldeRly: a study protocol for the C-SENIoR, a pragmatic non-randomized controlled trial
Rationale	Population ageing is one of the greatest social and economic challenges across European Union. Proton pump inhibitors (PPI) are a class of medications that reduce acid secretion and are used for treating many gastroesophageal conditions as reflux disease (GERD), dyspepsia, reflux esophagitis, peptic ulcer disease, etc. There is evidence that PPI are being overprescribed worldwide. About 25% to 80% of people are being prescribed with a PPI inappropriately. Potentially inappropriate medication (PIM) is defined as a medication in which the risk of an adverse event outweighs its clinical benefit. Specially for older people, chronic PPI use without clear indication contributes to polypharmacy and puts people at risk of experiencing drug interactions and adverse events at long-term (e.g., *Clostridium difficile infection*, pneumonia, hypomagnesaemia, and fractures). Successful deprescribing may result in reduced medication burden, ADEs and costs. Although it is unclear if withdrawal of medicines may significantly improve patients' quality of life. The adequate evaluation and quantification of benefits is also necessary to understand if the intervention costs are worth it from an economic viewpoint.
Aim and Objective(s)	The aim of this study is to evaluate the effectiveness and the cost-effectiveness of a community pharmacist-general practitioner collaborative deprescribing intervention of proton-pump inhibitors among the community dwelling elderly.<br><br>**Primary Objective:**<br>To evaluate the effectiveness of a community pharmacist-general practitioner collaborative intervention on the discontinuation of inappropriate PPI by community dwelling elderly.<br>**Secondary Objective:**<br>To assess the intervention impact on medication burden (total medicines) and on prescription optimization (drug-drug interactions); to assess time to PPI discontinuation; to understand if the intervention affects participants' beliefs with respect to inappropriate medicines and self-reported medication adherence; to evaluate the intervention implementation through process indicators; and to assess patients' satisfaction with the collaborative intervention.

	**Exploratory Objective:**<br>To explore factors (sociodemographic characteristics, clinical history, beliefs, others) potentially associated to patient's medication discontinuation.
Study Design	This is a pragmatic, multicenter, non-randomized controlled trial with a follow-up period of 6 months of a collaborative community pharmacist-general practitioner deprescribing intervention. There are two-arms in this parallel-trial: the collaborative intervention arm and the control arm (usual care). Patients will be recruited through community pharmacies.<br>An Economic evaluation will be conducted alongside the trial, meaning the collection of costs and outcome data alongside the trial.
Study setting	This study will be carried out in the primary care setting involving community pharmacies and Family Health Units (FHUs) in Portugal mainland.<br><br>FHUs' collaboration for the intervention arm development was established with two FHUs from Viana do Castelo region (FHU Uarcos and FHU Terra da Nóbrega). All pharmacies located in the same region/ municipality of the FHUs, that fulfilled the following criteria will be invited to participate (≈9 pharmacies):<br>• with Sifarma® dispensing software and Farmalink® data network installed.<br>• with registered prescription sales from identified FHUs.<br><br>For control arm, to avoid contamination bias, different geographic regions were chosen. Control geographical selection involved the identification of the best-matching to intervention settings firstly in terms of municipality sociodemographic characteristics and secondly in terms of FHU and its patients' characteristics (PPI consumption). It is planned to enroll more than 2 municipalities in the Control arm to guarantee a similar number of participating pharmacies, as a lower pharmacy recruitment rate (about 20%) is expected in this group compared to the Intervention group. Control pharmacies were identified based in the same criteria used for intervention pharmacies. All the identified pharmacies will be invited to participate (≈60).
Study Population	The study population comprises community-dwelling older adults with a continuous use (>8-week use) of PPI, recruited from community pharmacies.<br>**Inclusion Criteria**<br>Subjects must meet all the following criteria to be eligible for inclusion in the study:<br>• Age ≥ 65 years old<br>• Having a contact telephone number

	• Taking at least one PPI (POM or OTC) continuously for >8 weeks: omeprazole, lansoprazol, esomeprazol, pantoprazol, rabeprazole, as per International Non-Proprietary Names (A02BC, Anatomical Therapeutic Chemical (ATC) classification)<br>• Registered at the selected FHU (Uarcos and Terra da Nóbrega) in the intervention group and the identified FHUs on the following municipalities (Baião, Campo Maior, Elvas, Mondim de Basto, Penela, Ponte de Lima, Póvoa do Lanhoso, Reguengos de Monsaraz, Sobral de Monte Agraço, Vial Real, Vila Verde) on the control arm as self-reported by patients.<br>**Exclusion Criteria**<br>Individuals who do not give their informed consent, live in nursing homes or assisted-living facilities, are unable to communicate or speak in Portuguese, have any cognitive impairment, or any other condition that does not allow them to understand the study objectives or the questionnaire completion as perceived by the pharmacist, will be excluded from the study.
Intervention and Control	The intervention consists of a multidisciplinary collaborative care framework between intervention pharmacies and FHU to firstly deprescribe inappropriate PPI and secondly to identify and solve other related medicines-safety problems such as drug-drug interactions. The patient-centered multifaceted intervention package has several components: a). Pharmacists patients recruitment and assessment of the potential inappropriate use of PPI considering the patient self-reported clinical indication; b) Pharmacist direct-to-patient oral and written delivered educational information; c) Patients' therapeutic profile drafting and identification of relevant drug-drug interactions (DDIs) and medication duplications; d) Exchange of information regarding the PPI use and medication profile, between the pharmacist and the General Practitioner (GP); e) GP assessment of the patient PPI use and other medication related information; f) GP-patient consultation to discuss the PPI deprescribing pertinence and strategy and, solve other possible medication related problems (all patients will be contacted by the GP preferably by telephone, a face-to-face consultation can be scheduled if the GP considers relevant); g) Exchange of information between the GP and the pharmacist, regarding PPI withdrawal decision and adopted strategy agreed with the patient; h) Patient's follow-up by the pharmacist at 2 and 4 weeks after the GP reported data on PPI withdrawal decision, to monitor possible symptoms relapses and if applicable, define with the patient a control-symptoms strategy (non-pharmacological) pre-agreed with the GPs under the designed intervention. The 4-week telephone interview will occur only on patients with PPI withdrawal indication. Patients can at any moment seek advice with community pharmacies and/or general practitioners.

	This project will have the assistance of a clinical research associate (CRA) who will guarantee the information change flow foreseen between pharmacies and FHUs in the intervention arm.<br>Patients recruited in the pharmacies assigned to the control group receive usual care.<br>All the community pharmacists (intervention and control) will be trained by the research team in the study procedures.
Outcomes	The outcomes are measured at patient level.<br>The primary outcome is the successful deprescription – discontinuation or decreased dose of PPI, defined as a statistically significant reduction in medication burden between the intervention and control group between baseline, 3 and 6-month follow-up.<br>Secondary outcomes are:<br>• Time to PPI discontinuation considered the time until complete withdrawal since recruitment; Change in medication burden that is, the mean number of regular medications patient take and proportion of patients on polypharmacy (patients with 5 or more medicines) assessed at baseline and 6-month follow-up;<br>• Change in Drug-drug interactions, assessed at baseline and 6-month follow-up;<br>• Change in Adverse drug events, that is, the absolute and relative counts of self-reported adverse drug events and type of events (e.g., needed professional support) experienced by patients, assessed at baseline and 6-month follow-up;<br>• Change in Patient Reported Outcomes Measures (PROMs) assessed at baseline and 6-month follow-up: Quality of life related to health, will be evaluated using the five-level version of the European Quality of Life-5 Dimensions questionnaire (EQ-5D-5L instrument); Self-reported adherence using a 7 items Measure Treatment Adherence questionnaire (MTA); and Patients' beliefs about inappropriate medicines, assessed by the beliefs about medicines questionnaire (BMQ-specific) this assessed at baseline and 3 months;<br>• Satisfaction with the collaborative intervention (general and health professional related), at 6-month follow-up in the intervention group.<br>• Process outcomes, to assess the fidelity and quality of the collaborative intervention.
Participant Timeline	This trial will have an estimated recruitment period of two months, followed by a follow-up period of 6 months per patient. However, the recruitment period length will be redefined after the first month of the recruitment period, if needed.<br>Recruitment is expected to start in October 2022 (±4 months). Study 6-month follow-up is expected to end in March 2023 (±4 months).
Statistical Methods	A statistical analysis plan (SAP) will be drafted before any analysis takes place.<br>The null hypothesis proposes no difference in primary outcomes between intervention and control patients. An intention-to-treat population will be

	considered, including patients regardless to the degree to which they have been exposed to the intervention (as this is a pragmatic trial). Outcomes will be estimated for the whole dataset. 95% confidence intervals will be reported.<br>The description of the baseline characteristics including the self-rated health status (SRH), the primary and secondary endpoints will be presented for all patients and stratified by arm and other subgroups (eg.: age group, etc.). Comparisons between arms will be performed using the chi-square/Fisher test for categorical variables and/or t-test/ANOVA or nonparametric Wilcoxon/Kruskal-Wallis test for continuous variables. Significance level of 5% will be adopted.<br>The primary outcome of the study will be calculated using a GLM model for binary outcome with an identity link function to estimate the risk difference or the difference between Intervention and Control groups in the proportion of patients who discontinued or decreased PPI dosage at 6 months of follow up. Results will be adjusted for baseline covariates. Relative risk will be calculated as well as the number needed to treat (NNT) – the inverse of the difference in absolute rate of discontinuation between the intervention and control groups. Analysis will also be conducted at 3 months of follow up. Significance level adopted is $\alpha = 0.05$.<br>Regarding secondary outcomes, descriptive statistics will be calculated for all patients and reported with respect to each time point. For each outcome, adequate GLM models will be used to compare groups with respect to therapeutic outcomes (PPI specific and other medication), Quality of life (EQ-5D-5L), BMQ specific applied to PPI, Adherence (MAT) and healthcare resource utilization. Changes over time points will be evaluated.<br>Patients' level of satisfaction and process outcomes will be analyzed only for the intervention arm. Process outcomes and will be used to assess the fidelity and quality of the collaborative intervention.<br>Time to PPI discontinuation will be accessed through Kaplan-Meier (KM) estimator. Results will be stratified by group and KM curves will be presented. Log-rank test will be computed to compare results between cohort subgroups. In alternative, multivariate Cox Proportional Hazards models will be used if groups are unbalanced and hazard ratios computed.
Sample Size	Our study will be conducted to detect a minimal 20% increase in the rate of discontinuation of inappropriate medication use caused by the intervention compared with the usual care. Assuming an alpha of 5%, 90% power and an allocation ratio of 1:1, we estimate that the minimum total sample size to detect differences between intervention and Control group is 178 patients (89 patients per group).<br>Considering a 20% lost to follow-up, then a total of 222 patients (111 per group) will be needed. The sample size was calculated using the software G*Power version 3.1.9.6.

Economic Evaluation	An Economic Evaluation will be conducted alongside the non-randomized controlled trial, including the collection of costs and outcome data alongside the trial.<br>The economic evaluation will adopt a National Health Service perspective as base case, including all health care resource use data, but also a broader perspective in scenario analyses, including out-of-pocket expenses. The time horizon in the base-case analysis will be of 6 months.
Ethics/Legal	This study will comply with all requirements stated in the Portuguese Law of Clinical Investigation, the Declaration of Helsinki, and any other applicable local regulations.

2. Amendments and updates

Table 1 – Study protocol amendments and updates regarding previous approves by CEFCM-UNL

Number	Date (dd/mm/YYY)	Section of study protocol	Amendment or update	Reason
1	01/07/2022	6.6 Participant timeline	Start and End of study data collection	Because of COVID-19 we had to extend the beginning of the start of the study data collection. Additionally, there was a need to change the study geographic region from the Centre to the North region of Portugal which delayed the beginning of the study.
2	01/07/2022	5. Study design<br>6.1. Study setting	The geographic region, FHUs and pharmacies where the study will be conducted	Because the first region considered (Aveiro) to conduct the study, had a similar intervention running in the primary care units, we needed to change the intervention region. Currently, we aim to conduct the study in Alto Minho (ACES Alto Minho) region.<br>New intervention and control FHUs and pharmacies were identified and included.<br>The details of the intervention region and control FHUs were updated accordingly.
3	01/07/2022	6.2. Pharmacy invitation / recruitment	Pharmacies	Because eligible study pharmacies are those within the geographical area of the identified FHUs, new pharmacies had to be identified.
4	01/07/2022	6.3.1 Patients' inclusion criteria	Intervention and control FHU listed as an inclusion criterion	The characteristics of the new intervention and control FHUs were updated according to the geographical change
5	01/07/2022	9.1. Research ethics approval	Inclusion of a new Ethics Committee assessment (CES-ULSAM)	Because the study will be conducted in Alto Minho, an ethical assessment of the CES-ULSAM is required

Milestones

Table 2 - Planned dates of study milestones

**Milestone**	**Planned date**
Study Protocol and Privacy Impact Assessment	December 2020
Ethics Clearance	February 2021(CEFM-UNL)
Pharmacist and physicians training	September / October 2022 (±4 months considering Ethics approval)
Start of data collection	October 2022 (±4 months considering Ethics approval)
End of data collection	When full sample size estimate is reached with additional 6-month follow-up per patient (Estimation: March 2023 ±4 months)
Final report of study results	To be defined
Manuscript submission	To be defined

3. Background and Rationale

Population ageing is one of the greatest social and economic challenges across European Union (EU). The number of elderly people in the EU-28 rose, over the last two decades, at a rate that was almost six times as fast as for the overall population (1). In Portugal, currently, elderly account for more than one fifth of the total population, one of the highest shares in the EU. It is expected to exceed one-third by 2050 (1). Despite a positive increase in life expectancy, many face multiple health conditions or mobility problems in their last years (2). A recent study found that multimorbidity (presence of two or more chronic conditions) was present in 92.6% (CI:90.7-94.6) of people with 65 or more years attending primary care in Portugal (3).

Studies show that medicine consumption has increased in recent years (4,5), with older people being the largest per capita consumers (6). According to national data, the National Health Service (NHS) per capita medicines consumption was 15.8 in 2015 and increased to 16.5 in 2018 (7). Almost half of the medicines are used by people with more than 80 years old (8), with prescription medicine expenditures becoming particularly high for older adults (9). For example, in the USA, the average annual expenditures for people aged 80 and older are almost 1.5 times higher than those for people aged 50 to 64 years ($560 vs $796, respectively)(10).

Polypharmacy is widespread and increasingly common, driven by this growth of ageing population, increasing prevalence of multimorbidity and evolution in the complexity of therapeutics (11–14). As people get older there are also physiological changes, which impact the pharmacodynamics and pharmacokinetics of medicines, changing the benefit/risk ratio of medications. Approximately 1 in 5 medicines commonly used in older people may be inappropriate (15). Medications may be appropriate when initiated for an indication, but insufficient monitoring and follow-up, or even transfer between providers and care settings, can contribute for continued use to be inappropriate, with an outcome ranging from none (or poor) therapeutic benefit to severe harm and possible death(16). Globally, in 2017 the World Health Organization (WHO) refers that the cost associated with medication errors has been estimated at US$ 42 billion annually (17).

Potentially inappropriate medication (PIM) is defined as a medication in which the risk of an adverse event outweighs its clinical benefit, particularly when there is a safer or more effective alternative therapy for the same condition, so that its use should be avoided (18).

PIM has been identified as one of the main risk factors for adverse drug events (ADEs) in the elderly (19–22). PIMs can lead to a loss in quality of life, greater health care service use and, consequently higher health care cost among older adults (23–25). A study conducted in Canada found that patients presented to the Emergency Department (ED) with ADEs experienced a higher risk of additional in-patient days (6.3% versus 3.4%; odds ratio 1.52; 95% CI 1.43 to 1.62) and a higher rate of outpatient health care encounters (1.73 versus 1.22; rate ratio 1.20; 95% CI 1.03 to 1.40), compared with patients presenting for other reasons. The adjusted median monthly cost of care was 1.90 times higher (Can $325 versus $96; 95% CI 1.18 to 3.08) (26). In Europe, 8.6 million unplanned hospitalisations occur every year due to ADRs (27), with twice as many patients aged 65 years and older being hospitalized than their younger counterparts (28). However, ADEs can be difficult to recognize in older people and be attributed to a specific medicine, as they often take several medicines and present nonspecific symptoms, which may have several etiologies like fatigue, falls, or osteoporosis (29). Preventing harm due to medicines is a global patient safety challenge (17) that can improve health and reduce demands for the healthcare system reducing threats to service sustainability. However, General practitioners (GPs) face major challenges to balance benefits and risks. Besides lack of time to manage polypharmacy (30), they also need to coordinate prescribing from multiple specialists.

Patient safety is one of the most important components of health care delivery. Deprescribing has been proposed as a method of reducing polypharmacy and improving medication use throughout a patient's path of care. It is a structured process of reduction or discontinuation of inappropriate medication that might be causing harm or might no longer be providing benefit, supervised by a health care professional (31–33). Evidence supporting the benefits and safety of deprescribing has growing in recent years (34,35). A recent review (36) concluded that successful deprescribing interventions are facilitated by the combination of behavior change techniques (BCTs) involving a range of intervention components such as defining a goal and an action plan to solve inappropriate prescription, clear instructions and guidance, information about potential risk and consequences of deprescribe or not deprescribe. Various studies also identified the necessity and benefits of multidisciplinary collaboration when addressing polypharmacy (37). Ideally the withdrawal plan should be developed in a cooperative process between the patient, the physician and the community pharmacist, followed by a close monitoring during, and after medication withdrawal (33,38–40).

Uncertainty and avoidance of harm as adverse drug withdrawal events return of medical condition(s), reversal of drug-drug interactions and damage to the health professional-patient relationship, has

been commonly reported as a barrier to deprescribing. However, research has shown that community dwelling elderly are willing to undertake deprescribing, especially if they take a large number of medications, are experiencing side effects or feel some medications are no longer necessary (41–44). Moreover, evidence suggests that patients with multimorbidity incur high levels of out-of-pocket expenditures (OOPE) on medicines and that the elderly and low-income groups were most vulnerable to higher OOPE (45). In Portugal, this issue is very relevant specially when there are problems of access to health care (46,47). The reduction or withdrawal of unnecessary medicines, by reducing OOPE, can also encourage patients to follow the health professional indication. The need to receive health professional support and follow-up by various means, if a medication was discontinued, and the insurance that the medication could be restarted, if necessary, a major factor of success (42,43,48).

Many studies reported a high prevalence of PIM in European community-dwelling older adults (20). National research has also showed a high prevalence use of inappropriate medicines among elderly across Portugal (49–51). Simões *et al* found that PIM was present in 68.6% (95% CI 65% to 72%) of older adult population within primary care in Portugal (51). These results suggest there is an opportunity to act and deprescribe. Medicines inappropriately prescribed or used for too long, can contribute to polypharmacy with its attendant risks of nonadherence, prescribing cascades drug interactions, medication errors, emergency department visits, and hospitalizations (52).

Proton pump inhibitors (PPI) are a class of medications that reduce gastric acid secretion and are used for treating many conditions such as gastroesophageal reflux disease (GERD), dyspepsia, reflux esophagitis, peptic ulcer disease (PUD), and as part of the eradication therapy for *Helicobacter pylori* (*H. pylori*) infection (53). They are also used to prevent and reduce the risk of ulcers in people with a history of PUD and in people who use chronic nonsteroidal anti-inflammatory drugs (NSAIDs) (54). Although short-term use of PPI appears relatively safe, long-term use as been associated with *Clostridium difficile* infection, hip fracture, chronic kidney disease (CKD) diarrhea, impaired B12 absorption, hypomagnesaemia, or community-acquired pneumonia (55–59). Yet, studies consistently show that PPI are being overprescribed worldwide (60–62). Evidence suggests that adverse effects from long-term use of PPI are increasing, and that older people are more susceptible to them (63). Treatment should be given at the lowest effective dose that controls symptoms, and for the minimum period (64). It is estimated that between 25% to 80% of people are prescribed a PPI inappropriately (31,65–67), being frequently reported as one of the most prescribed class of PIM among the elderly. This inappropriate use incurs a financial burden to the patient and high costs to the health system. In

2016, the PPI' costed to the Portuguese NHS €19.9 million and €37.9 million to patients (68). Pantoprazole, a common PPI, was among the top 10 most consumed medicines in ambulatory care in Portugal in 2019. Pantoprazole market sales increased by 5.8% compared to 2018 (69). Therefore, it is prudent to discontinue unnecessary PPI.

PPI are frequently addressed in diverse available screening tools used to identify PIMs in older adults (70–73) Beers Criteria, one of the most known and used criterion in the world (74), strongly recommend, with high-quality evidence, to avoid scheduled use of PPI for >8 weeks except for high-risk patients (eg, oral corticosteroids or chronic NSAID use), erosive esophagitis, Barrett esophagitis, pathological hypersecretory condition, or demonstrated need for maintenance treatment (eg, because of failure of drug discontinuation trial or H2-receptor antagonists) (71). Despite the existence of these tools and national guidelines to help health care professional to improve prescription, inappropriate use of these medicines persists. PPI were one of the most frequently class of PIMs found in Simões *et al* observational nationwide study (51). Furthermore, this class of medicines in the lower doses is classified as over the counter (OTC) drug, which makes their access easier for patients.

Successful deprescribing may result in reduced medication burden, ADEs and costs (34), although it is unclear if withdrawal of medicines may significantly improve patients' quality of life (QoL) or mortality (35,75).

Due to limited resources, patients who may benefit from deprescribing need to be prioritized. Qualitative research has shown that not only patients are receptive to make changes to their PPI (76) but also health professionals pointed this class of medications as a priority to deprescribe supported by evidence-based guidelines (77). Communication and deprescribing efforts shared between physicians, pharmacists, and patients, may enhance the inappropriate medication withdrawal process keeping it safe and maintaining the patient trust (78). Multidisciplinary collaborations to deprescribe in primary care seem to be successful to optimize prescription and medication use in different settings and models(79–81). Despite these interventions and collaborations are potentially costly and time consuming, few studies addressing the cost-effectiveness of trials to reduce PIM have been conducted (82–84)

In Portugal healthcare is mostly provided in a very fragmented way, both between primary and hospital care within the NHS and, between the NHS and the private sector like the community pharmacy one. Indeed, these sectors are organized and financed in different and independent ways, and the

incentives for collaboration are mostly absent. Using this knowledge and for the purpose of this study we will consider a collaborative model, as the establishment of formal links between community pharmacies and primary care units, with the aim of improving medicines use among elderly in Portugal.

The C-SENIoR (Collaborative Deprescribing Intervention of PPI on community dwelling elderly) trial aims to determine whether a general practitioner and community pharmacist collaborative deprescribing intervention targeting community-dwelling elderly- is effective and cost-effective compared to usual care.

4. Aim and objectives

The aim of this study is to evaluate the effectiveness and the cost-effectiveness of a community pharmacist-general practitioner collaborative deprescribing intervention of proton-pump inhibitors among the community dwelling elderly.

Primary objective

To evaluate the effectiveness of a community pharmacist-general practitioner collaborative intervention on the discontinuation of inappropriate PPI by community dwelling elderly, compared with usual care, as measured by the cessation or reduction of targeted medication at 6 months follow-up. The acronym C-SENIoR stands for “Collaborative depreScribing intervENtion of PPI on community dwelling eldeRly”.

Secondary objectives

To assess the intervention impact on medication burden (total medicines) and on prescription optimization (drug-drug interactions); to assess time to PPI discontinuation; to understand if the intervention affects participants’ beliefs with respect to inappropriate medicines and self-reported medication adherence; to evaluate the intervention implementation through process indicators, and to assess patient’s satisfaction with the collaborative intervention.

Exploratory objectives

To explore factors (sociodemographic characteristics, clinical history, beliefs, others) potentially associated to patient’s medication discontinuation.

5. Study Design

This is a pragmatic, multicentre, non-randomised controlled trial with a follow-up period of 6 months of a collaborative Pharmacy-Family Health Unit deprescribing intervention. There are two-arms in this controlled trial: the intervention arm (collaborative intervention) and the control arm (usual care). Pragmatic trials are undertaken in the real world under the context of a real-life setting and the results are intended to support a decision to deliver the intervention in a routine practice (85).

An experimental design with random assignment was not feasible since the collaborative intervention was developed based on the expressed interest of two specific family health units (FHUs) – FHU Terra da Nóbrega in Ponte da Barca municipality and FHU Uarcos municipality in Arcos de Valdevez (both belonging to Aces Alto Minho, Unidade Local de Saúde do Alto Minho). Randomization at patient level was not considered to prevent individual contamination, due to controls and interventions being exposed to the same environment (pharmacies and FHUs). Each FHU unit and surrounding pharmacies will consist of a study site (intervention or control).

The Ponte da Barca and Arcos de Valdevez sites will comprise the intervention arm. Control sites will be selected from other geographical regions with characteristics like the intervention study sites.

Patients will be recruited through community pharmacies. A 1:1 ratio (intervention:control) of participants will be used. The flowchart of the C-SENIoR trial is illustrated in Figure 1.

An Economic evaluation will be conducted alongside the trial, meaning the collection of costs and outcome data alongside the trial.

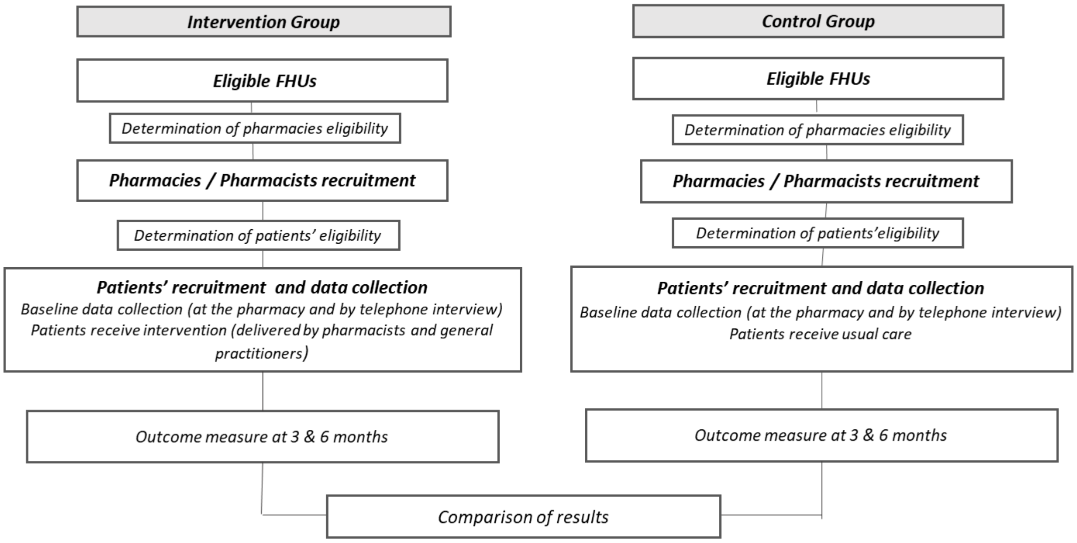

Figure 1 - Study Flowchart

6. Methods

PARTICIPANTS, INTERVENTIONS, AND OUTCOMES

6.1. Study setting

This study will be carried out in the primary care setting involving community pharmacies and Family health Units (FHU) in Portugal mainland.

FHUs' collaboration for the intervention arm development was established with two FHUs from the Health Centres Group - ACES Alto Minho/ULSAM in Viana do Castelo district, which showed interest to participate in a community pharmacist-general practitioner collaborative trial in primary care. The ACES Alto Minho (AM) comprises a geographic region of ten municipalities in the north region of Portugal with about 232 178 inhabitants in 2017: Arcos de Valdevez, Caminha, Melgaço, Monção, Paredes de Coura, Ponte da Barca, Ponte de Lima, Valença, Viana do Castelo, Vila Nova de Cerveira. Viana do Castelo is one of the most aged districts in Portugal. In 2018, the ACES AM Ageing Index was 220,3% and the old age dependency rate 39.2%, well above the mainland average (162.2% and 22.9 respectively). Moreover, the illiteracy rate (6.8%) was above the mainland average. For the 2016-18 triennium, the life expectancy at birth was 80.81 years, which is very similar to the national value (80.99) (86).

Form the intervention arm, pharmacies who fulfilled the following criteria will be invited to participate (≈9 pharmacies):

- Be affiliated in ANF (which represent 95% of the Portuguese community pharmacies).
- Location in the same municipality of the identified FHUs.
- With Sifarma® dispensing software (≈84% of all community pharmacies), to allow researchers to extract electronic data, on the acquisition of medicines by patients.
- With registered prescription sales from identified FHU

It is expected that the proportion of pharmacies accepting to participate in the intervention arm will be high (about 80% of the total invited) since this is an innovative health care service, involving multidisciplinary teams and collaborative interventions delivered by professionals from FHUs and Pharmacies. The recruitment strategy will be reinforced by several initiatives (e.g., telephone contacts

made by the principal investigator to the technical director of the invited pharmacies) to engage the participation of pharmacies.

To avoid contamination bias, different geographic regions were chosen for the recruitment of control pharmacies and patients. The control selection involved the identification of the best-matching to intervention setting characteristics, firstly in terms of municipality sociodemographic characteristics and secondly in terms of existence of FHUs with similar traits of the intervention FHU (type and PPI consumption).

The following criteria was used to identify control regions for intervention study sites:

1) Municipalities with most similar characteristics of intervention sites (Arcos de Valdevez e Ponte da Barca), as estimated by three-dimensional normalized Euclidean distance, considering:
    - Municipalities *per capita* Purchasing Power (PP) ('Índice de Poder de Compra Concelhio per capita', 2019) (INE, 2022)
    - Municipality Aging ratio (AR) ('Índice de Envelhecimento no Concelho', 2020) (INE, 2022)
    - Municipality Illiteracy Rate (IR) ('Taxa de analfabetismo (%) por Local de residência', 2011) (Pordata, 2020)
2) FHUs from identified municipalities considering:
    - Contractual NHS type - Type "A" or "B" as the intervention units, as governance and funding models differ from FHU type (A or B).
    - Prescription volume of PPI per 1000 elderly (≥65 y) in 2021, as FHU age pyramid (2022, data extracted in June 2022 from the site BI-CSP) most similar with the FHUs intervention. It was accepted a maximum absolute variation of 20% in comparison with the estimated value of the FHU intervention.

Additionally, it is planned to enroll more municipalities in the control arm to guarantee a similar number of participating pharmacies, as a lower pharmacy recruitment rate is expected in this group compared to the intervention. We expect an average pharmacy participating rate of about 20% (about ¼ of the expected rate of the Intervention group) as previous found in others non-interventional studies conducted in pharmacies (source: CEFAR).

After identification of the FHUs, control pharmacies will be identified based on the same criteria used for intervention pharmacies. All the identified pharmacies will be invited to participate (≈60). Detailed information of the intervention and control municipalities and FHUs are depicted in Table 3.

Table 3 - Details of the intervention and identified municipalities and control FHUs

Group	FHU name and Type	N.elderly Registered at FHU	N. GP (BI-CSP, 2022)	Municipality / District
Intervention	Uarcos - B	3546	7	Arcos de Valdevez / Viana do Castelo
	Terra da Nóbrega - A	3573	10	Ponte da Barca / Viana do Castelo
Control	Senhora da Graça – B	1911	11	Mondim de Basto / Vila Real
	Penela -B	1910	4	Penela / Coimbra
	Baião – B	1812	6	Baião / Porto
	Lethes – B	3436	9	Ponte de Lima / Viana do Castelo
	Mais Saúde - B	2071	5	
	D'As Terras de Lanhoso – B	2797	7	Póvoa de Lanhoso / Braga
	Prado – B	12339	6	Vila Verde / Braga
	Pró-Saúde - B	6976	5	
	Vida+ - B	12152	7	
	Nova Mateus - A	1889	8	Vila Real / Vila Real
	Fénix – A	3266	7	
	Corgo - A	3111	6	
	Raia Maior – A	2043	5	Campo Maior / Portalegre
	Costa Campos – A	1527	2	Sobral de Monte Agraço / Lisboa
	Remo – A	2901	6	Reguengos de Monsaraz / Évora
	Amoreira – A	3310	8	Elvas / Portalegre
	Uadiana - A	2052	6	

General practitioners (GP); Family Health Units (FHU)

6.2. Pharmacy Invitation / Recruitment

A personalized invitation will be sent to the eligible community pharmacy owners in the geographical areas of the identified FHUs. Community pharmacies that intend to participate will be asked to fill in a participation form listing the participating pharmacists, correspondent pharmaceutical society number, and the name of the pharmacist(s) who will be responsible for the study implementation and procedures of the study in the community pharmacy.

The community pharmacies which agree to participate (intervention and control) will be trained in the study procedures by the research team (for more detail see sub-section “training”).

Each community pharmacy will be asked to recruit at least 10 patients.

6.2.1. Retention Strategies

To maximize retention of pharmacies and pharmacy staff, follow-up phone calls and regular recruitment achievements email reports will be sent by the study research team to each pharmacy.

6.3. Study population and Eligibility criteria

The study population comprises community-dwelling older adults (≥ 65 years old) with a long-term use (>8-week use) of any PPI, recruited from community pharmacies.
PPI medication was chosen for this trial, based on its high consumption in Portugal (PPI was among the top 10 more consumed medicines in ambulatory care on Portugal in 2019) and among these population (51), availability as OTC medicines allowing an easier access to them, health professionals report of PPI as one of the preferential groups of medicines to deprescribe (77), and on the following high-evidence criteria / recommendations:

- 2019 Updated Beers Guidelines for Inappropriate Prescription (45)
- The EU (7)-PIM list (46)
- Portuguese PPI- Directorate-General for Health clinical guidance (87)

All inclusion and exclusion criteria will be followed by the community pharmacies’ staff to ensure that the patient is eligible for the study (**Annex 1-**enrollment guide). If a patient is eligible, the pharmacist

will explain the aim of the study, invite the patient to participate and sign the Informed Consent form (ICF) (**Annex 2**), before any intervention and/or data collection.

Patients who fulfill the inclusion criteria, have no exclusion criteria, agree to participate, and sign the ICF will be enrolled consecutively for a two-month period (can be extended if needed to achieve the sample size). Both groups (intervention and control) will undergo a 6-month follow-up study period.

6.3.1. Individuals' inclusion Criteria

Subjects must meet all the following criteria to be eligible for inclusion in the study:

- Age ≥ 65 years old
- Having a contact telephone number
- Taking at least one PPI (POM or OTC) continuously for >8 weeks - omeprazole, lansoprazol, esomeprazol, pantoprazol, rabeprazole, as per International Non-Proprietary Names (A02BC, Anatomical Therapeutic Chemical (ATC) classification).
- Registered at the selected FHUs as self-reported by patients:
    - In the intervention group - FHU Uarcos (Arcos de Valdevez) or Terra da Nóbrega (Ponte da Barca) and,
    - In the control arm - FHUs listed on table 3 from the identified control municipalities.

6.3.2. Exclusion Criteria

Individuals who do not give their informed consent, live in nursing homes or assisted-living facilities, are unable to communicate or speak in Portuguese, have any cognitive impairment, or any other condition that does not allow them to understand the study objectives or the questionnaire completion as perceived by the pharmacist, will be excluded from the study.

6.4. Study Enrollment and Patient Recruitment Procedures

During the recruitment study period, a pop-up window will be generated on participating pharmacies' software Sifarma® whenever a PPI is dispensed in the pharmacies (intervention and control), so that the pharmacist will be warned to systematically assess individual's enrollment eligibility criteria. Eligible individuals will be invited to participate in the study.

The patient can refuse to participate, and the pharmacist will be instructed to fill in the refusal form with basic sociodemographic information (e.g gender, age group) and motives for refusal (**Annex 3**). If accepts to participate, he/she will be required to sign an informed consent before any data collection or intervention procedure.

The research team will monitor the pharmacies (intervention and control) through regular phone calls, to maximize patient enrollment and reach the target sample size. During the recruitment period a feedback report containing major key performance indicators (e.g., number of recruited patients) will be provided to pharmacies.

6.5. Intervention and control

The intervention consists of a multidisciplinary collaborative care framework between intervention pharmacies and FHU to firstly deprescribe inappropriate PPI and secondly to identify and solve other related medicines-safety problems such as drug-drug interactions. The patient-centered multifaceted intervention package has several components.

Firstly, following in-person patient eligibility assessment, recruitment, acceptance and informed consent signature, the pharmacist will collect study information regarding the PPI medicine use and beliefs, through the application of a structured paper-based questionnaire (**Annex 4).** Pharmacist will then assess the potential inappropriate use of PPI, considering the patient self-reported clinical indication for it use and the Deprescription algorithm (**Annex 5).** Following pharmacist will inform the patient about the screening result (unknown, possible recommendation to maintain the PPI or, possible recommendation to withdraw based in patients' self-report) and that he/she will be contacted by the research team in the following 48-72h for additional data collection, and by the GP in 1 to 2-week to assess the medication. Furthermore, the pharmacist will deliver a direct-to-patient oral and written educational information, in the shape of a booklet (**Annex 6)**, concerning the rational use of medicines, PPI correct indications and safe use, deprescribing benefits and possible withdraw symptoms.

Secondly, after patient recruitment, a therapeutic profile will be drawn up and analyzed by the research team to list all medications taken by the patient in a regular basis and identify medication duplications and relevant drug-drug interactions (DDIs)[1] (**Annex 7**). The therapeutic profile will be drafted based in extracted data from the pharmacies software regarding the sales associated to the patients' tax information number (NIF) and confirmed by patient self-report, via a telephone interview with the research team. DDIs will be assessed using Sifarma® software. In this telephone interview, the research team will confirm the use of the listed medications (prescription-only-medicines (POMs) and OTC). Further data for study purposes (e.g PROMs) will also be collected in the telephone interview (**Annex 8**). The final therapeutic profile and related safety information will then be sent to the community pharmacist by email (with access key) to be shared with the patient GP for clinical assessment.

Thirdly, the pharmacist will gather all the information, the PPI deprescribing recommendation and the therapeutic profile with identified moderate and severe DDIs provided by the research team and share with the GP. A specific paper case report form "Passaporte do utente" (**Annex 9**) will be used to exchange information between both pharmacists and GP.

Fourth, The GP with the information provided by the pharmacist and that in his/her medical records, will assess the PPI use and other reported safety issues (DDIs). The patient will be contacted by the GP to discuss the PPI deprescribing pertinence and strategy and, solve other possible medication related problems. All patients will be contacted by the GP preferably by telephone. A face-to-face consultation can be scheduled if the GP considers relevant. Fifth, the PPI withdrawal decision and adopted strategy agreed with the patient will be communicated to the pharmacist using the paper case report. Sixth, after the GP report to pharmacist, of the PPI withdrawal decision, pharmacist will contact the patient by telephone at 2 and 4 weeks, to monitor possible symptoms relapses, answer questions and if applicable, define with the patient a control-symptoms strategy (non-pharmacological) pre-agreed with the GPs under the designed intervention. The 4-week telephone interview will occur only on patients with PPI dose reduction or withdrawal indication. At the end of the withdrawal follow-up

[1]We intend, as part of the intervention, to be the pharmacist to collect all information regarding patients' medication profile. However due to the Covid-19 pandemic, the research team thought to reduce patient-community pharmacist face-to-face interaction time, and withdrawal from the pharmacist intervention package the task not directly related with the main PPIs' deprescribing and educational goals.

period, pharmacist will share the follow-up information with the GP using the established communication model. If any severe symptom is identified during this time span, pharmacists can send an urgent alert email to the FHU research coordinator. To assist the pharmacist through all intervention, a complete intervention log form, which include the telephone interview script **(Annex 10),** will be made available by the research team.

Patients can at any moment seek advice with community pharmacies and/or general practitioners. The overview of the multifaceted collaborative intervention package is depicted in Figure 2. intervention details are accessible on the C-SENIoR Intervention Flowchart (**Annex 11)**.

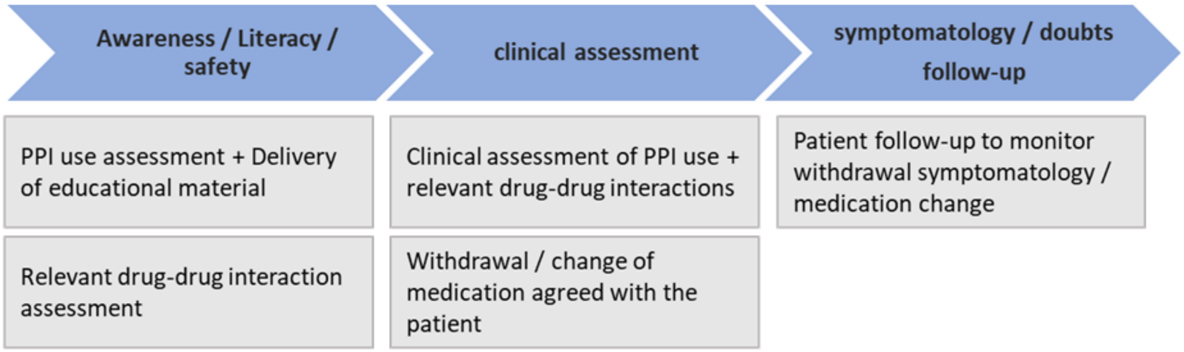

Figure 2 - Overview of the C-SENIoR multifaceted collaborative intervention package

The educational booklet was developed by the research team (pharmacists and general practitioners) for the study purpose. We performed a literature review of resources already tested in deprescribing interventions and available online (e.g., https://deprescribing.org/) and followed Fajardo et al recommendations (88) concerning readability of patient education materials for deprescribing interventions. The booklet is being tested with PPI elderly users, regarding its readability, dimension, key messages comprehension and images understanding.

This project will have the assistance of a clinical research associate (CRA) who will guarantee the information change flow foreseen between pharmacies and FHUs in the intervention arm.

Patients recruited from pharmacies assigned to the control group will receive usual care. For both groups, a baseline paper-based questionnaires will be delivered by the pharmacist (Annex 4), and three

telephone interviews, will be conducted by the research team at baseline, 3- and 6-month follow-up, respectively, to collect study data (see table 1).

Training

Before initiation of the study, both control and intervention pharmacists will receive training regarding study procedures or study and intervention procedures, respectively.

Control group pharmacists will be provided a training session to explain the study procedures focused on the eligibility criteria, enrolment strategy and questionnaires application. The intervention group pharmacists will also receive specific training on physiopathology changes in the elderly, deprescribing risks and benefits, intervention, and study procedures. The training sessions will be provided by the research team (2 pharmacists - Sónia Romano and Isabel Guerreiro both with an Educational Training for Trainers certificate and 1 general practitioner) and will be delivered in webinar format of a 2 to 4-hour session. The training is mandatory to participate in the project. All intervention (this part just for intervention arm) and study materials will be delivered to the pharmacies by express mail. The trainers will not receive any payment for the preparation and training sessions.

A training session will also be delivered to all general practitioners of the FHUs intervention arm, mostly focused on the study procedures (e.g., aim, population, intervention design, schedule, materials), and Portuguese PPI- clinical guidance. The training will be delivered by the FHU coordinator, the CEFAR research team contact person, in an FHU internal meeting.

The research staff will be available to answer questions and provide support as necessary, to all community pharmacists and general practitioners from the intervention harm enrolled in the study.

6.6. Participant timeline

This trial will have an estimated minimum recruitment period of two months, followed by a follow-up period of 6 months per patient. However, the recruitment period length will be redefined after the first month of the recruitment period, if needed.

Recruitment is expected to start in October 2022 (±4 months). Study 6-month follow-up is expected to end in March 2023 (±4 months) (base case).

6.7. Study Follow-up

Study follow-ups include 2 telephone calls, and electronic data extraction from the pharmacies dispense software at 3- and 6-months post recruitment. Telephone interviews are expected to last 10-15 minutes.

6.8. Outcomes

The outcomes are measured at patient level. Patient's outcomes data will be collected at baseline and at study follow-up points (3 and 6 months). Follow-up data can be collected within 1 month after or before these exact time-point. At baseline, data collection is performed through a face-to-face interview conducted by the pharmacist (before the intervention take place in the intervention arm), by electronic extraction from the pharmacies dispense software and by telephone interview led by the research team after the patient recruitment. Follow-up study data collection is conducted by the research team through patients' telephone interview and electronic extractions from the pharmacies dispense software. Intervention related data will be collected alongside the study.

For process outcomes and economic evaluation analysis, data will be collected from the intervention log form (**Annex 10**), from the case report form "Passaporte do utente" (**Annex 9**) developed for the information exchange between pharmacists and GP, and from the CRA monitoring form (**Annex12**). These materials will be collected for study purposes in the end of the intervention period.

Primary outcomes

The primary outcome is successful deprescription – discontinuation or decreased dose of PPI, defined as a statistically significant reduction in medication burden between the intervention and control group between baseline and 3- and 6-months follow-up ascertained by pharmacy medicine sales associated to the patients' NIF and confirmed by patient self-report.

Based on Deprescribing meaning (31), discontinuation of a PPI will be defined as:

- the lack of reported consumption assessed at 3 and 6-month follow-up, as well as the absence of initiation of another medicine from the same class, or;

- the reported dose-reduction assessed at 3 and 6-month follow-up, as well as the absence of initiation of another medicine from the same class.

The discontinuation/dose reduction rate among participants in the intervention arm will be compared to the discontinuation/dose reduction rate among participants in the control arm. In this way we will be able to determine the absolute rate of discontinuation attributable to the intervention.

Secondary outcomes

Time to PPI discontinuation

- Survival analysis of PPI discontinuation, considered the time until complete withdrawal since recruitment (Kaplan-Meier method);

Change in other Medication-related parameters | Profile and Safety outcomes.

- Medication burden that is, the mean number of regular medications patient take and proportion of patients on polypharmacy (patients with 5 or more medicines as commonly accepted definition (89)), assessed at baseline and 6-month follow-up.
- Drug-drug interactions (DDIs), that is, the absolute and relative counts of drug-drug interactions and by degree of severity, patients with any drug-drug interaction and with severe interactions. Most frequent severe drug-drug combinations will also be reported. Drug-drug interactions will be assessed by sifarma® software interaction alert system considering all medications taken, assessed at baseline and 6-month follow-up. Moderate and serious DDIs will be checked by the researchers using the Sifarma® software safety module. This module is an Electronic Information platform on medicines integrated on the Sifarma® dispense software, with updated information regarding clinically relevant Drud-Drug interactions (DDIs). This platform is powered with the summary of medicine characteristics (RCM) and other information sources as Stokley's Drug Interactions and Lexicomp. DDIs are classified according with 2 types of severity: a) Moderate DDI - Drug associations which benefit from (or for which it is recommended) monitoring, dose adjustment or any other form of management; b) Severe DDI - potential life-threatening and/or requiring medical intervention to minimize or prevent serious adverse effects; drugs are contraindicated for concurrent use. The software

displays an automatic alert regarding the DDIs identified and provide additional information regarding the mechanism of interaction.

- Adverse drug events, that is, the absolute and relative counts of self-reported adverse drug events and type of events (e.g., needed professional support) experienced by patients, assessed at baseline and 6-month follow-up.

Change in Patient Reported Outcomes Measures (PROMs):

- Quality of life related to health, will be evaluated using the five-level version of the European Quality of Life-5 Dimensions questionnaire (EQ-5D-5L instrument) (90,91), assessed at baseline and 6-month follow-up; EQ-5D-5L scores will be computed for each patient.
- Patients' beliefs about inappropriate medicines, assessed by the beliefs about medicines questionnaire (BMQ-specific)(92) applied to PPI; assessed at baseline and 3-month follow-up. It comprises two sub-scales: the specific necessity scale (SNS) assessing patients' views on their personal need for their medication and the specific concerns scale (SCS) assessing patients concerns towards their medication.
- Adherence using a self-reported 7-item Measure Treatment Adherence (MTA) tool (93), applied at baseline and 6-month follow-up. The MTA, a psychometric tool derived from Morisky et al, evaluates the individuals' behaviour in relation to the daily use of medication. Each item is scored from 1 (always) to 6 (never). Points are summed and divided by the total number of items. Higher scores indicate higher self-reported adherence. Patients with values of 5 and 6 are considered adherents. We select this method to measure multiple medication adherence because is simple to apply and was validated for the Portuguese Population in 2001, with a reported Cronbach's alpha of 0.74.

Patient reported experiences measures (PREMs)

- Satisfaction with the collaborative intervention (general and health professional related), measured by a 5-point Likert scale at 6-month follow-up in the intervention group.

Process outcomes, will be measured only in the intervention arm and will be used to assess the fidelity and quality of the collaborative intervention:

- Proportion of GP replies to the initial report of the pharmacy;

- Number and type of pharmaceutical opinions sent by the pharmacist to the GP;
- Number and type of GP decisions related to PPI deprescribing and safety data;
- Proportion of concordant pharmacist opinion-GP deprescribing decision;
- Proportion of patients who experienced the follow-up telephone contact by the pharmacist accordingly to the intervention protocol and;
- Health professional's compliance with predefined intervention steps times.

Table 4 illustrates a summary of time points and parameters measurements in both trial arms and process measurements collected only in the intervention arm for fidelity and quality analysis.

Table 4 - Overview of data collection schedule and measurements

Time points / Data	**T0**	**Ongoing** (only intervention arm)	**T1**	**T2**
	Recruitment - 0	Intervention time span	3 months	6 months
Socio-demographic and clinic characterization	X			
Self-rated health status (SRH)(94)	X			
PPI specific outcomes	X		X	X
Other medication related outcomes	X			X
Quality of life (EQ-5D-5L)(90,91)	X			X
BMQ specific(92) applied to PPI	X		X	
Adherence (MTA)	X			X
Healthcare resource utilization (Economic Evaluation)	X			X
Intervention satisfaction questionnaire				X
Process parameters		X		

6.9. Blinding

Due to the nature of the intervention, neither patients nor pharmacist and GPs (providers) are blinded to the intervention. In addition, research team who developed this trial cannot be blinded either. However, to preserve a certain level of masking, to protect sources of bias, intervention participants will only be aware that the research team will also collect data in other geographical regions regarding usual care.

In the control group, no information regarding the intervention procedures will be provided to pharmacies and patients. The project will be presented as a medication use pattern study in the elderly. Moreover, as pharmacies allocated to each group (intervention and control) are located in distinct geographic locations, minimizing their interactions and ensuring their unawareness of each other.

FHUs in the control sites will be blind by design as they do not have any intervention in this trial, serving only to ensure that patients in both arms are subject to the same type of usual care.

6.10. Potential harms and patient safety

Because of the pharmacological characteristics of the PPI there is a possibility that the discontinuation might lead to symptoms relapse (e.g., rebound hyperacidity) or complications (95). However, the potential for serious harm due to adverse drug withdrawal events (ADWEs) appears to be rare (96) and several drug class specific deprescribing interventions have found no difference in the rate of ADWEs between control and intervention groups (35). We expect a low probability of relapses or complications as only non-evidenced based medications will be withdrawn and the best evidence deprescribing guidelines will be followed (87). Additionally, patients will have the pharmacist and general practitioner support to help them to manage potential symptomatology with non-drug approaches, use of OTC medicines or, if necessary, to review the PPI dose, indication to use on-demand or full restart of the medication based on the best evidence. Any suspected adverse drug event (ADE) acknowledged will be reported to the study research team and to the National Pharmacovigilance System thru though “Portal RAM” from the National Authority of Medicines and Health Products, I.P (INFARMED) site.

6.11. Sample size

Our principal hypothesis is that our intervention will reduce the use of inappropriate PPIs compared to usual care.

Our assumption is that the intervention will reach a rate of discontinuation similar with that achieved in previous studies reporting medication withdrawals in intervention with collaborative work between pharmacists and GP, compared to usual care (minimum absolute difference of 20%)(79,97). Alongside,

we will also assume that a small rate of discontinuation will be observed in the group without the intervention (control Group) as much as of 10%.

Therefore, our study will be conducted to detect a minimal 20% increase in the rate of discontinuation of inappropriate medication use caused by the intervention compared with the usual care. Assuming an alpha of 5%, 90% power and an allocation ratio of 1:1, we estimate that the minimum total sample size to detect differences between intervention and control group is 178 patients (89 patients per group).

Considering a 20% lost to follow-up, then a total of 222 patients (111 per group) recruited in the participating pharmacies will be needed in this study.

The sample size was calculated using the software G*Power version 3.1.9.6.

Exact - Proportions: Inequality, two independent groups (Fisher's exact test)

**Options:**	Exact distribution		
**Analysis:**	A priori: Compute required sample size		
**Input:**	Tail(s)	=	Two
	Proportion p1	=	0.1
	Proportion p2	=	0.3
	α err prob	=	0.05
	Power (1-β err prob)	=	0.90
	Allocation ratio N2/N1	=	1
**Output:**	Sample size group 1	=	89
	Sample size group 2	=	89
	Total sample size	=	178
	Actual power	=	0.9017447
	Actual α	=	0.0340267

Monthly sales data retrieved from the pharmacies' system (hmR database®) shows that in 2021 and YTD:May22, the number of packages sold every month per pharmacy of PPI is about 340 in this municipalities and is rising. This number is 16.2 % lower in Arcos de Valdevez (320) than in Ponte da Barca (380).

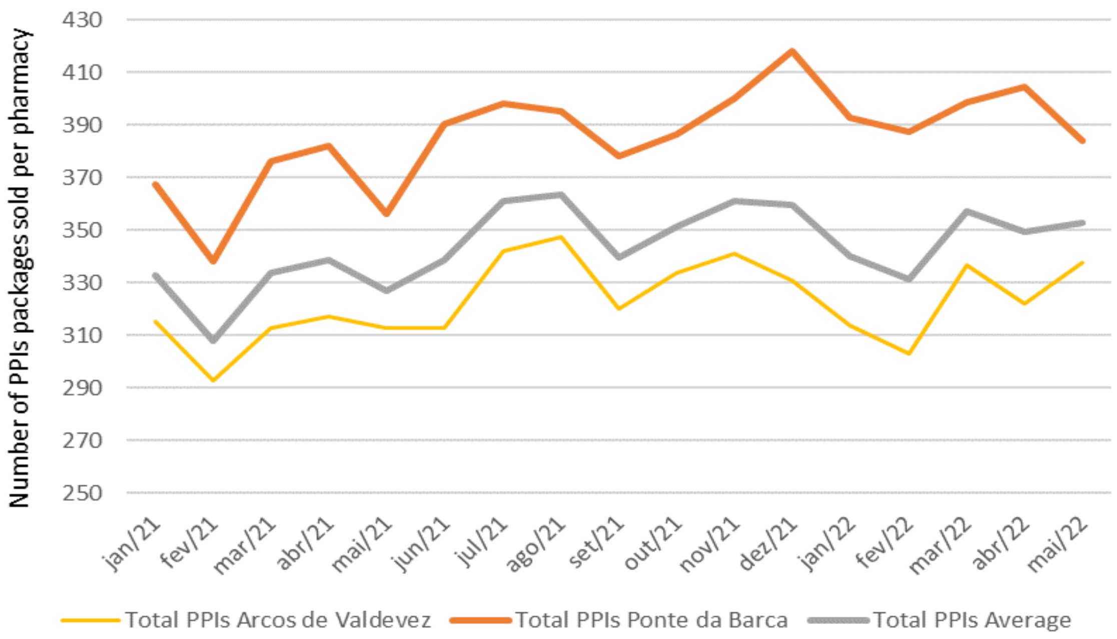

Figure 3 - Average number of PPI sales per pharmacy in intervention Municipalities Jan 21-mai 22 (hmR sell-out data)

On the other hand, official data collected from FHU BI reports 2021 indicates that, on average, in the 2 intervention FHUs, about 70% (72.4% in Arcos de Valdevez and 68.5% in Ponte da Barca) of PPI prescriptions were for elderly patients (65 years and older), suggesting that most all prescribed PPI sales could be potential eligible patients to be recruited for the study, with respect to the age criteria.

Analyzing routinely dispense data from the Pharmacies' software (June 2022/CEFAR), we observed that about 94% of the sales of PPI that were prescribed in these two FHU were sold on the pharmacies selected to participate in the study. This shows not only a very good regional PPI prescriptions (FHU) and sales (pharmacies) concentration, but also, in a more general term, that patients from these FHUs tend to acquire their medication locally. This is a good indication that once the recruitment is started, the number of potential eligible patients to invite for the study is high with respect to the pair FHU – Pharmacy criteria.

One thing that is important to notice is that only less than 1% of all PPI currently dispensed in these pharmacies are sold without a prescription (OTC medicines). This may include acute usage which is not eligible for entering the study. In this study the recruitment invitation will proceed to all eligible

patients carrying or not a prescription refill of PPI. This suggests that overall monthly PPI sales data could be a good starting point in the defining the recruitment period.

If the refusal rate would range between 50% and 75%, then it is expected that the recruitment period could last from 1 to 2 months to reach the estimated minimum sample size of the study.

DATA COLLECTION, MANAGEMENT, AND ANALYSIS

6.12. Data collection, Variables and Definitions

Data will be collected through:

a. Structured paper-questionnaire applied to patients by face-to-face interview;

b. Structured questionnaires applied to patients by telephone interview;

c. Data extractions of medicines consumption from the pharmacy dispensing software and;

d. Paper forms filled by the pharmacists and general practitioners during the intervention course.

e. Paper form filled by the CRA during the intervention course.

Patient outcomes data will be collected at all study-follow-up points.

Follow-up data for T1 and T2 could be collected within 1 month after or before these exact time-points.

The personal data of the participants (name, telephone number, NHS and NIF) will be collected to enable the patient identification by the health professional and to assure that the research team have timely and appropriate access to essential data to answer the primary objective.

Table 5 summarises the list of variables, time points and data sources that will be collected for this study.

Table 5 – List of variables

Variable name	Operationalization	Data source	Time point
**Sociodemographic**			
FHU_name	Text	Informed consent	T0
NHS_number	9-digits	Informed consent	T0
NIF number (tax information number)	9-digits	Informed consent	T0
Name	text	Informed consent	T0
Telephone	9-digits phone number	Informed consent	T0

Sex	• Male<br>• Female	Paper-based questionnaire	T0
Date of birth	• dd/mm/yyyy	Informed consent	T0
Employment status	• retired<br>• working part-time<br>• Working full time or<br>• Other	Telephone interview	T0
Household dimension	• 1 (alone)<br>• 2<br>• 3 to 4<br>• 5 or more<br>• Number children under 18y	Telephone interview	T0
Educational level (highest completed)	• None<br>• I – Basic education (4 years)<br>• II – Basic education (6 years)<br>• III – Basic education (9 years)<br>• Secondary education or equivalent (12 years)<br>• Bachelor/University Degree	Telephone interview	T0
Household average monthly income (after the discounts have been made). Consider all the sources of income as pension, family monetary support, rents, subsides, etc	• Menos de 400 euros<br>• + 400 até 800 euros<br>• + 800 até 1.200 €<br>• + 1.200 até 2.000 €<br>• + 2.000 até 3.000 €<br>• + 3.000 até 5.000 €<br>• + 5.000 €	Telephone interview	T0
Medicines reimbursement scheme	• General<br>• Special (e.g pensioners with higher reimbursement rates)<br>• Other.________	Paper-based questionnaire	T0
**Clinical and anthropometric Characteristics**			
BMI	• Weight (kg)<br>• Height (cm)	Paper-based questionnaire	T0

Smoking status	0=Never smoked<br>1=Ex-smoker<br>1.1 Ex-smoker <12 months<br>1.2 Ex-smoker >12 months<br>2=Current smoker<br>2.1. Daily<br>2.2. Less than daily	Telephone interview	T0
Comorbidities *(based in the SHARE study*(98) *and ICHOM older person reference guide*(99)*)*	• A heart attack including myocardial infarction or coronary thrombosis or any other heart problem including congestive heart failure<br>• High blood pressure or hypertension<br>• High blood cholesterol<br>• A stroke or cerebral vascular disease<br>• Diabetes or high blood sugar<br>• Asma<br>• Chronic lung disease such as chronic bronchitis or emphysema<br>• Arthritis, including osteoarthritis (arthrosis, joint degeneration), or rheumatism<br>• Osteoporosis<br>• Hip fracture or femoral fracture<br>• Cancer or malignant tumour, including leukaemia or lymphoma, but excluding minor skin cancers (within the last 5 years)<br>• Parkinson disease<br>• Anxiety<br>• Depression<br>• Kidney disease<br>• Cataracts<br>Other.___________	Telephone interview	T0

**Medicine related measurements (profile and safety) (within the last 6 months -T0 and T2)**			
PPI use	• SKU identifier, INN, dosage, posology	Paper-based questionnaire/ pharmacy software / telephone interview	T0, T1, T2
PPI use duration	• n Time (month/years)	Paper-based questionnaire	T0
PPI use indication (self-reported)	• Do not know.<br>• GERD with esophagitis or without esophagitis but with persistent symptoms<br>• Peptic ulcer disease with recurrent episodes<br>• Severe esophagitis<br>• Zollinger-Ellison syndrome/ Barrett's esophagus<br>• Peptic ulcer prevention in long-term NSAID users<br>• GERD without esophagitis and symptoms<br>• mild and moderate esophagitis<br>• Dyspepsia without symptoms<br>• Peptic ulcer disease without symptoms<br>• Eradication for H. pylori bacteria<br>• Other	Paper-based questionnaire	T0
PPI initial Prescriber	• General Practitioner<br>• Gastroenterologist<br>• Other. _______________<br>• Do not know	Paper-based questionnaire	T0
PPI continuous use	• Yes<br>• No	Pharmacy software confirmed by telephone interview	T1, T2
Date of PPI discontinuation	• mm/yyyy<br>• Unknown/cannot remember	Telephone interview	T1, T2

Other regular Medicines use	• SKU identifier, INN, dosage, posology	Pharmacy software confirmed by telephone interview	T0, T2
Other regular Medicines use indication	• text	telephone interview	T0
Drug-Drug interactions (DDIs)	• Number and Type (moderate, severe)	Pharmacy software	T0, T2
Adverse drug events	• Yes<br>• No	Telephone interview	T0, T2
Number of Adverse drug events	• number	Telephone interview	T0, T2
Adverse drug events. Needed support from health professionals?	• Yes<br>• No	Telephone interview	T0, T2
**Healthcare resource utilization (within the last 6 months -T0 and T2**			
Number GP's appointments all	• n GP appointments	Telephone interview	T0, T2
Number of Specialty practitioners' appointments	• n medical speciality health care appointments (ex. internal medicine)	Telephone interview	T0, T2
Diagnostic tests or medical exams (gastric system related)	• Yes<br>• No	Telephone interview	T0, T2
Type of Diagnostic tests or medical exams (gastric system related)	• To be defined	Telephone interview	T0, T2
Number Diagnostic tests or medical exams (gastric system related)	• n diagnostic tests or exams	Telephone interview	T0, T2
Diagnostic tests or medical exams with anesthesia	• n	Telephone interview	T0, T2
Emergency room (ER) visits Y/N	• Yes<br>• No	Telephone interview	T0, T2
Number of Emergency room (ER) visits	• n emergency room visits	Telephone interview	T0, T2
Hospitalizations Y/N	• Yes<br>• No	Telephone interview	T0, T2

Hospitalizations - Name of the Hospital	• text	Telephone interview	T0, T2
Number of Hospitalizations (total)	• n hospitalizations	Telephone interview	T0, T2
Number by Type of Hospitalization	• n hospitalizations unplanned<br>• n hospitalizations planned	Telephone interview	T0, T2
Major motive for Hospitalization	• text (e.g. organ/ surgery)	Telephone interview	T0, T2
Hospitalization LOS	• n (nights)	Telephone interview	T0, T2
Hospitalization LOS ICU	• n (nights)	Telephone interview	T0, T2
Private setting healthcare resource use (hospitalizations, medical appointments, etc)	• Yes<br>• No	Telephone interview	T0, T2
Type and OOPE expenses	• Type (eg. ER visit) and value (€)	Telephone interview	T0, T2
**Patient reported outcomes Measures (PROM)**			
Quality of Life health related	• EQ-5D-5L	Telephone interview	T0, T2
Beliefs	• Patients' Beliefs about Medicine Questionnaire (BMQ)	Paper-based questionnaire / Telephone interview	T0, T1
Self-rated health status (SRH)	• Very Bad<br>• Bad<br>• fair<br>• Good<br>• Very Good	Paper-based questionnaire	T0
Self-reported Adherence	• MTA Questionnaire	Telephone interview	T0, T2
**Patient reported experience measures (PREM) (only in the intervention group)**			
Satisfaction with the baseline educational pharmacist-led intervention	• likert scale from 1 to 5 – not satisfied at all to very satisfied	Telephone interview	T2
Satisfaction with the overall pharmacist intervention	• likert scale from 1 to 5 – not satisfied at all to very satisfied	Telephone interview	T2

Satisfaction with the overall GP intervention	• likert scale from 1 to 5 – not satisfied at all to very satisfied	Telephone interview	T2
Satisfaction with the global collaborative intervention	• likert scale from 1 to 5 – not satisfied at all to very satisfied	Telephone interview	T2
**General Practitioners /pharmacists intervention process (only in the intervention arm)**			
Number GP appointments Intervention related	• n GP appointments	Telephone interview	T2
Number GP appointments intervention related reported to pharmacists	• n GP appointments	Paper form	ongoing
GP appointments date	• *dd/mm/yyyy*	Paper form	ongoing
GP appointments reported to pharmacists_Type	• Face-to-face<br>• Telephone<br>• Other	Paper form	ongoing
GP_ PPI deprescribe decision	• Indication to deprescribe<br>• Indication to maintain<br>• Indication to reduce the dose<br>• Other	Paper form	ongoing
Pharmacy_baseline_interview_ date	• *dd/mm/yyyy*	Paper-form	ongoing
Pharmacy_baseline_interview_ Duration	• Time (minutes)	Paper-form	ongoing
Pharmacy_PPI screnning	• recommendation to deprescribe<br>• recommendation to maintain<br>• Inconclusive	Paper-form	ongoing
Pharmacy GP report_preparation_Baseline	• Time (minutes)	Paper-form	ongoing
Pharmacy-patient_telephone_follow-up_date	• dd/mm/yy	Paper form	ongoing

Duration of the patients' interview plus time spent in the preparation (minutes)	• Time (minutes)	Paper form	ongoing
**Other data**			
ANF pharmacy code	5-digit code	Pharmacy's software / Paper-based questionnaire	T0, T1, T2
FHU name	• Text (name)	Informed consent	T0

T0- baseline; T1 – 3 months; T2 – 6 months.

6.13. Data management and Quality control

The filled patient-paper questionnaires, intervention paper forms and other data collection tools can be sent by mail or deliver in-person to the CRA that then will make the deliver to the research team.
The database for the insertion of the questionnaires and other formularies data will be created specifically for the purpose of this study and tested to check for data integrity. Researchers will assure database quality processes are followed for completeness and accuracy, and in accordance with the following data validation plan: database will not allow the duplication of records. Electronic databases will be stored on a secure shared computer drive only accessible by study researchers and all paper forms collections will be kept in locked cabinets.
To ensure that no patient is enrolled twice in the study, variables regarding date of birth, gender and NHS number will be checked for duplicates. Only authorized personnel from within the research team will have access to patient or provider personal data. A unique patient study ID will be assigned, which will consist of the first letter of the person's first and last name, and their date of birth in the format DD/MM/YYYY. When this ID is not available, the NIF unique numerical identifier can be used as provided by patient.
Quality control procedures will be used to check data collection and storage. Data storage will be checked and compared with the information written on the paper questionnaires for a random sample of questionnaires, in accordance with the Acceptance Quality Level (AQL) (100). Patients' inclusion criteria will be double checked (pharmacy and research team). All errors will be corrected. Further checks for possible inconsistency in information will be performed and appropriate corrections will be made.
Study researchers are responsible to ensure that the study is conducted, and data are generated, documented, and reported in compliance with the protocol, accepted standards of Good Pharmacoepidemiology Practice, and all applicable legislation, rules and regulations relating to the conduct of the study.

All data protection procedures are further detailed in the Privacy Impact Assessment (PIA) of this study, complying with the requirements of the GDPR.
All instruments and questionnaires to be applied in the study are detailed in the supplemental material section of the protocol (annexes).

This research is conducted by CEFAR/infosaúde, the research center of the National Pharmacy Association (ANF), which has interests in this topic. The support by academic independent researchers, who do not receive any fees for collaboration, will guarantee that these interests do not interfere in the analysis and interpretations. These conflicts of interests will obviously be declared in all scientific publications.

6.14. Data recording and document retention

CEFAR must maintain source documents for each patient in the study, consisting of data collected at the pharmacy (including dispensing records), at the FHU by general practitioners and by telephone interview by the research team, containing demographic, PROs and clinical information.

6.15. Statistical Methods

A statistical analysis plan (SAP) will be drafted before any analysis takes place.

6.15.1. General principles

The null hypothesis proposes no difference in primary outcomes between intervention and control patients.

Descriptive statistics will be calculated for the full dataset and study arm. Categorical outcomes will be summarized by absolute and relative counts, including counts of missing observations. Continuous outcomes will be summarized by the number of non-missing values, mean, standard deviation (SD), median, lower and upper quartiles and minimum and maximum values.

The description of the baseline characteristics including the self-rated health status (SRH), the primary and secondary endpoints will be presented for all patients and stratified by arm and other subgroups (e.g., age group, etc.).

Comparisons between groups will be performed using the chi-square/Fisher test for categorical variables and/or t-test/ANOVA or nonparametric Wilcoxon/Kruskal-Wallis test for continuous variables. Significance level of 5% will be adopted.

6.15.2. Study outcomes

An intention-to-treat population will be considered, including patients regardless to the degree to which they have been exposed to the intervention (as this is a pragmatic trial). Outcomes will be estimated for the whole dataset. 95% confidence intervals will be reported.

Primary outcomes

Primary outcome of the study will be calculated using a GLM model for binary outcome with an identity link function to estimate the risk difference or the difference between Intervention and Control groups in the proportion of patients who discontinued or decreased PPI dosage at 6 months of follow up. Results will be adjusted for baseline covariates. Relative risk will be computed as well as the number needed to treat (NNT) – the inverse of the difference in absolute rate of discontinuation between the intervention and control groups. Analysis will also be conducted at 3 months of follow up. A 1 year analysis can be conducted to measure the maintenance of the intervention effect. Significance level adopted is $\alpha = 0.05$.

Secondary outcomes

Descriptive statistics will be calculated for all patients and reported with respect to each time point. Adequate GLM models will be used to compare groups with respect to clinic characteristics, therapeutic outcomes (PPI specific and other medication), Quality of life (EQ-5D-5L), BMQ specific applied to PPI, Adherence (MTA) and healthcare resource utilization. Changes over time points will be evaluated.
Patients' level of satisfaction and process outcomes will be analyzed only for the intervention arm. Process outcomes will be used to assess the fidelity and quality of the collaborative intervention.

Time to PPI discontinuation will be accessed through Kaplan-Meier (KM) estimator. Results will be stratified by group and KM curves will be presented. Log-rank test will be computed to compare results between cohort subgroups. If groups are unbalanced with respect to potential confounders, multivariate Cox Proportional Hazards models will be used instead. Univariate and multivariate hazard ratios (HR) will be computed, and Wald's 95% CI presented. Patients will be censored in the survival

analysis if they were lost to follow-up (subjects who could not be reached by telephone interview, or the primary outcome could not be computed or withdrew consent), hospitalized or died.

6.15.3. Enrolled population and refusing subjects.

Screening data will be collected on the population of pharmacies and patients invited to participate in this study. We will report:

a) the total number of pharmacies in the study area, the total number of eligible pharmacies invited to participate, the total number of pharmacies who accept to participate and frequency of reasons for refusal if available. We will compare pharmacy characteristics (number of staff, rural/urban, volume of dispenses) of participating and non-participating pharmacies and test for significant differences using chi-square test for contingency tables.
b) the total number of patients invited to participate, the total number of patients who accept to participate and frequency of reasons for refusal if available. Age (group), gender, household dimension and higher educational level completed of the patients who are invited and refuse to be included in the study and of the patients enrolled will be tested for significant differences using chi-square test for contingency tables.

6.15.4. Population of analysis

Primary analysis will only consider patients for which a 6-month reported PPI use is available (here the definition of a complete case), assuming the reports are missing at random (MAR) if not available. Nevertheless, MAR assumption will be tested comparing patients with missing 6-month information regarding primary outcome with the remainder sample of patients with respect to the baseline characteristics.

6.15.5. Timing of analysis

No interim analyses are planned. Analysis will take place after the last patient completed the 6-month assessment.

6.15.6. Statistical software

Data analysis will be performed using SAS and/or R software.

6.16. Site monitoring

Formal site monitoring will be performed by an CRA, and principal researcher related to data collection and intervention compliance. Researchers will also give previous training to the pharmacists and general practitioners to conduct the project according to the protocol.

6.17. Economic evaluation

6.17.1. Overview

Economic evaluation will be conducted alongside the non-randomized controlled trial, meaning the collection of costs and outcome data alongside the trial.

The economic evaluation will adopt an NHS perspective as base-case, including all health care resource use data and medicines costs, but also a broader perspective in a scenario analysis, including out-of-pocket expenses.

Data will be presented by costs parcel to allow alternative perspectives to be considered separately. The time horizon in the base-case analysis will be of 6 months.

6.17.2. Measurement and valuation of resources

Health care resource use data will be based on patient-reported data. Data will be collected for the following period: baseline and 6-month follow-up. At baseline, information will cover the previous 6 months; at 6-month follow-up the service use since the previous time-point was recorded (baseline).

Resource-use information will be collected using questionnaires developed by the research team. The following resources, identified on literature (84,101) will be collected: regular medications used, number of doctor’s appointments by type and setting (GP and specialist, public and private); number of emergency care episodes, number and major motive of hospitalizations, length of stay (LOS), number and type of diagnostic and therapeutic tests related with gastrointestinal symptoms.

For each item of service use reported in the resource use questionnaires, a unit cost will be applied and the total costs for each individual will be calculated. All unit costs will be for the most recent financial year over which the trial data will be collected and will be reported in Euros. Unit costs will be retrieved mostly from Portuguese official sources.

6.17.3. Intervention costs

The cost of delivering the intervention to participants (e.g. training of pharmacy and FHU staff, number and type of GP's appointments, time spend by the pharmacist delivering the intervention alongside the time spent in preparing it, undertaking administrative or other miscellaneous tasks) is obtained from the research team, from the pharmacists records filled at each patient intervention point (Annex 10) and, from patient self-reported data collected during the telephone interview (e.g. GP appointment intervention related) compared against the information recorded by the GP on the "patient passport form" (Annex 9) and valued using established methods (102).

6.17.4. Outcomes for the economic evaluation

6.17.4.1. Primary outcome

Cost-effectiveness will be explored in terms of the primary outcome measure (the proportion of patients who discontinued or decreased PPI dosage in the 6-month assessed by pharmacy medicine sales associated to the patients' tax information number (NIF) and confirmed by patient self-report).

6.17.4.2. Health-related quality of Life

The Health-Related Quality of Life (HRQoL) will be assessed using the EQ-5D-5L questionnaire applied at baseline and 6-month follow-up. The EQ-5D-5L is a generic preference-based measure of health state that covers five dimensions (mobility, self-care, usual activities, pain/discomfort, and anxiety/depression) that can be rated according to five levels of severity to define unique health states. The five-level version has showed increased reliability, sensitivity (discriminatory power) and feasibility compared to the previous three-level instrument. Participants are classified into one of 3125 health states, each associated with a score that can be used to calculate quality adjusted life years (QALYs) (103)

HRQoL weights for these health states have been previously elicited for the Portuguese population by Ferreira et al. (91,104). A score of 1 represents perfect health and a score of 0 represents death. The EQ-5D scores will be used to estimate patient-specific QALYs using the area under the curve method (105).

Mean QALYs measured by EQ-5D will be estimated using regression analysis to control for patients' covariates, including baseline EQ-5D score, (106) and will be used as a secondary outcome measure in the economic analysis.

6.17.5. Cost and cost-effectiveness analysis

Costs will be modelled with the most appropriate distribution for data. All analyses will be adjusted for pre-specified baseline characteristics, plus baseline costs.

Cost-effectiveness will be explored in terms of the primary outcome measure (reduction in PPI use) and QALYs and, will be assessed through the calculation of incremental cost-effectiveness ratios (ICER) – the additional cost of one intervention compared with another divided by the additional effects – using the net benefit approach (107). Cost-effectiveness acceptability curves will be presented by plotting the probability of the intervention being cost-effective for a range of possible values of willingness to pay for unit improvements in outcome (108). These curves are a recommended decision-making approach to dealing with the uncertainty that exists around the estimates of expected costs and expected effects associated with the interventions under investigation.

7. Study limitations

Methodologic limitations related to bias and confounding are inherent to the non-randomized design adopted in this study.

Recruitment and selection bias

- The intervention FHUs were selected based on willingness to develop a collaborative intervention and may not represent a generalized sample of FHUs population.
- The pharmacies are also selected based on willingness to participate and may not represent a random sample from any well-defined population.
- Positive selection bias of patients: Strategies to minimize patient's self-selection will be adopted, namely a pop-up reminder at the pharmacy software whenever a PPI is dispensed during the recruitment period. We will also compare the characteristics between the patients who are approached and refused to participate in the study and the patients enrolled in the study.
- Some patients may be lost to follow-up during the study period. Depending on the volume of dropouts and missing data which may bias the data collection and the results, the baseline characteristics of this sub-group will be compared with the baseline characteristics of all patients recruited for the study per arm to assess any selection bias.
- To determine whether control allocation was effective descriptive statistics (means, proportions) will be calculated to assess the balance between the groups on important confounders such as age, sex, health status, baseline beliefs about medications and the burden of medicines. If such confounders are unbalanced between the groups, we will produce unadjusted and adjusted estimates of the primary and secondary outcomes.

- As General practitioner usual practices can also be a confounding domain, control patients were selected from the population served from FHUs in the control municipalities, with PPI prescription patterns similar to the interventions FHUs, defined by the volume of prescribed PPI per 1000 elderly (≥65 y) in 2021, as FHU age pyramid.

Information bias

- Self-reported medication taken and clinical information. It is believed that the patients are aware of the duration of exposure to a specific medication and clinical indication for its use as also of her/his co-morbidities. A pharmacist from the research team will interview the patients and this will help on the validation of the different medicines taken and co-morbidities.
- Recall bias (e.g., consumption of healthcare resources). Measuring health care consumption through patient's self-report can be associated with a recall bias, however, collecting data from clinical/hospital records is not feasible. Additionally, in cases as hospitalization or urgency visit, we do not expect patients not to remember if an impactful event like these occurred in the last 6 months.

Generalisability

- Study sites are restricted to specific regions of Portugal and population. Results may not be applicable to overall individuals or settings.

8. Safety Reporting and Related Procedures

8.1. Adverse Events and Serious Adverse Events

An **adverse event (AE)** is generally defined as any unfavorable and unintended diagnosis, symptom, sign (including an abnormal laboratory finding), syndrome or disease which either occurs during the study, having been absent at baseline, or if present at baseline, appears to worsen. Adverse events are to be recording regardless of their relationship to the study intervention.

Changes resulting from normal growth and development that do not vary significantly in frequency or severity from expected levels are not to be considered adverse events. Examples of this may include, but are not limited to, teething, typical crying in infants and children and onset of menses or menopause occurring at a physiologically appropriate time.

A **serious adverse event (SAE)** is generally defined as any untoward medical occurrence that results in death, is life threatening, requires inpatient hospitalization or prolongation of existing hospitalization, results in persistent or significant disability/incapacity, or is a congenital anomaly.

Other important medical events that may not result in death, may not be life-threatening, or may not require hospitalization may be considered a Serious Adverse Event when, based upon appropriate medical judgment, they may jeopardize the patient or subject and may require medical or surgical intervention to prevent one of the other outcomes listed previously. Examples of such medical events include allergic bronchospasm requiring intensive treatment in an emergency room or at home and blood dyscrasias or convulsions that do not result in inpatient hospitalization.

8.2. Adverse Event Reporting

All AEs that occurred in enrolled patients exposed to the inhaler medication of interest or other concomitant medication, irrespective of seriousness or causality will be reported to the local Health Authority in accordance with national regulatory requirements for individual case safety reporting.

9. Ethics and dissemination

Complying with General Data Protection Regulation (GDPR), a Privacy Impact Assessment (PIA) was conducted following the terms below:

- Data sources used will be pseudoanonymized prior to treatment according to the personal data treatment procedures followed by the research team, developed under the scope of the

General Data Protection Regulation. Personal identification data will only be accessed for information insertion and/or completion.

- Collected personal information will be treated anonymously and confidentially, and no subject will be individually identified in any report or publication.
- All members of the research team in the study are aware of the General Data Protection Regulation and bound to proceed accordingly.
- All data transferred between parties will be checked regarding loss of information or data breaches according to the data transferring procedures implemented.
- All computer platforms and physical files ensure restricted access to information by the research team and investigators follow the implemented procedures for data processing and reporting of failures, developed in accordance with the General Data Protection Regulation. The destruction of information in paper support will be performed by a certified, third-party company, fulfilling the same regulation.
- Access to personal data, as well as opposition to treatment, rectification requests or erasure and limitation of treatment will be possible to any subject at any time. Delivery of personal data in a structured format or portability requests to another designated entity are also guaranteed. Subjects are also reserved the right to, at any time, withdraw their consent, leading to the immediate disposal of all personal information. All rights can be exercised by contacting the data treatment responsible for the study.

9.1. Research ethics approval

Complying with the Regulation (EU) 2016/679 of the European Parliament and of the Council of 27 April 2016 on the protection of natural persons with regard to the processing of personal data and on the free movement of such data, and repealing Directive 95/46/EC (General Data Protection Regulation), this study was firstly submitted to the Ethics Committee (EC) of the Nova Medical School, Faculdade de Ciências Médicas (CEFCM). Additionally, it will be submitted to the Comissão de Ética para a Saúde da ULSAM (CES-ULSAM).

Participants will have the right to withdraw fully or partially from the study at any time and for any reason, without jeopardizing patient service, by the general practitioner, pharmacist or at the pharmacy. All collected data will be strictly confidential.

The documentation to be sent for approval by the EC includes this protocol, all the relevant study materials, and the Informed Consent form.

This protocol was designed in accordance with the SPIRIT 2013 Statement (Defining Standard Protocol Items for Clinical Trials). It adheres to the ethical principles mandated by the 2008 Declaration of Helsinki and encompasses the protocol items recommended by the International Conference on Harmonisation Good Clinical Practice E6 guidance.

9.2. Informed consent procedures

In the context of proposed trial and data collection for the present study, an Informed Consent (written in Portuguese language) (**Annex 2**) will be obtained by the community pharmacist, from all patients in accordance with local practice and regulations. The informed consent will follow to IRB/ERC requirements, applicable laws, and regulations. Due to the study design, to minimize bias, two distinct informed consents forms will be applied – one to intervention arm (Annex 2.1) and other to the control arm (Annex 2.2.). The difference is mainly focused on the information regarding the intervention details.

Eligible patients may only be included in the study after providing written informed consent. The background, purpose, possible risk, benefit and procedures of the proposed study will be explained verbally and by written form to the patient. The participant shall keep a copy of his Informed Consent, which contains the contact of the research team, enabling, at any time, the request for clarification and non-participation. A copy will be left to the pharmacist and a third copy will be sent to the research team.

The participant is granted his / her rights to refuse or terminate study participation, privacy, information, and access to data, without penalty or changes in the normal access to health care.

9.3. Confidentiality of study/subject Data

The Subject ICF will incorporate wording that complies with relevant data protection and privacy legislation. Pursuant to this wording, subjects will authorize the collection, use and disclosure of their personal data by the Investigator and by those persons who need that information for the purposes of the trial and the study.

The Subject ICF will explain that study data will be stored in a computer database, maintaining confidentiality in accordance with the local law for Data Protection.

The personal identifiable data (patient´s name, tax number and phone number) will be collected for study extraction data and patients contact purposes. Data security measures will be taken to ensure the confidentiality and integrity of the information collected.

All collected data will be strictly confidential.

Data sources used will be anonymized prior to treatment according to the personal data treatment procedures, followed by the research team developed under the scope of the General Data Protection Regulation. Personal identification data will only be used by health care to exchange information between health professionals and for data collection by CEFAR research team in order to assess the primary and secondary objectives of the study.

9.4. Declaration of interests

This research is conducted by CEFAR/Infosaúde, the research center of the ANF, which has vested interests in this topic and funded the research.

Neither the pharmacists, nor the general practitioners or subjects enrolled in the study will be remunerated for their participation in the study. Additionally, the support by academic independent researchers, who do not receive any fees for collaboration, will guarantee that these interests do not interfere in the analysis and interpretations.

These conflicts of interests will obviously be declared in all scientific publications.

The research team guarantees that outcomes of the project will be submitted for publication, regardless of the positive, negative, or non-significant impact of the intervention.

9.5. Funding

This trial is funded by ANF/Infosaúde. No payments are made to researchers, FHUs, pharmacies or patients to conduct or participate in this study. Study design included in the protocol, as future publications of the results, is and will be from the exclusively responsibility of the research team.

9.6. Disseminating Policy

It is expected to communicate the research's potential contributions through publications in international and national peer-review journals, and in international and national meetings related to areas such as Public Health, Epidemiology, Health Economics and Outcomes Research.

Publications will comply with the International Committee of Medical Journal Editors (ICMJE) guidelines, which states: Authorship credit should be based on (1) substantial contributions to conception and design, acquisition of data, or analysis and interpretation of data; (2) drafting the article or revising it critically for important intellectual content; (3) final approval of the version to be published. Authors should meet conditions 1, 2, and 3. Conflicts of interest will be entirely displayed.

References

1. European Commission - Eurostat. People in the EU - statistics on demographic changes - Statistics Explained [Internet]. [cited 2020 Apr 15]. Available from: https://ec.europa.eu/eurostat/statistics-explained/index.php?title=People_in_the_EU_-_statistics_on_demographic_changes#Population_change_in_the_EU
2. European Commission - Eurostat. Ageing Europe - Looking at the lives of older people in the EU. Vol. 315, European Union. 2019.
3. Prazeres F, Santiago L. Prevalence of multimorbidity in the adult population attending primary care in Portugal: a cross-sectional study. BMJ Open. 2015;5(9):e009287.
4. Guthrie B, Makubate B, Hernandez-Santiago V, Dreischulte T. The rising tide of polypharmacy and drug-drug interactions: population database analysis 1995-2010. BMC Med. 2015;13:74.
5. Charlesworth CJ, Smit E, Lee DSH, Alramadhan F, Odden MC. Polypharmacy Among Adults Aged 65 Years and Older in the United States: 1988–2010. J Gerontol A Biol Sci Med Sci. 2015 Aug;70(8):989–95.
6. Linjakumpu T, Hartikainen S, Klaukka T, Veijola J, Kivelä SL, Isoaho R. Use of medications and polypharmacy are increasing among the elderly. J Clin Epidemiol. 2002;55:809–17.
7. INFARMED. Estatística do Medicamento e Produtos de saúde - 2018. Lisboa; 2019.
8. Ministério da Saúde (2018). Retrato da Saúde. Lisboa, Portugal; 2018.
9. Morgan SG. Prescription drug expenditures and population demographics. Health Serv Res. 2006 Apr;41(2):411–28.
10. Prescription Drugs | Health Policy Institute | Georgetown University [Internet]. [cited 2020 Jun 3]. Available from: https://hpi.georgetown.edu/rxdrugs/
11. Salisbury C, Edwards A, Steiner J, Main D, Schneider E, Morris M. Multimorbidity: redesigning health care for people who use it. The Lancet. 2012 Jul 7;380(9836):7–9.
12. Urzal J, Pedro AB, de Oliveira IF, Romero I, Achega M, Correia I, et al. Inappropriate prescribing to elderly patients in an internal medicine ward. Acta Med Port. 2019;32(2):141–8.
13. Kantor ED, Rehm CD, Haas JS, Chan AT, Giovannucci EL, HM K. Trends in prescription drug use among adults in the United States from 1999-2012. JAMA. 2015 Nov 3;314(17):1818–31.
14. Payne RA, Avery AJ, Duerden M, Saunders CL, Simpson CR, Abel GA. Prevalence of polypharmacy in a Scottish primary care population. Eur J Clin Pharmacol. 2014 May 1;70(5):575–81.
15. Roughead EE, Anderson B, Gilbert AL. Potentially inappropriate prescribing among Australian veterans and war widows/widowers. Intern Med J. 2007 Jun;37(6):402–5.
16. Pharmaceutical Group of the European Union. Patient safety. Maximizing patient safety in Europe through the safe use of medicines. Brussels: PGEU; 2007.
17. World Health Organization. Medication without harm - Global patient safety challenge on medication safety. World Health Organization. Geneva; 2017.
18. Corsonello A, Pranno L, Garasto S, Fabietti P, Bustacchini S. Potentially inappropriate medication in elderly hospitalized patients. Drugs Aging. 2009;26 Suppl 1:31–9.
19. Price SD, Holman CDAJ, Sanfilippo FM, Emery JD. Association Between Potentially Inappropriate Medications From the Beers Criteria and the Risk of Unplanned Hospitalization in Elderly Patients. Annals of Pharmacotherapy. 2014;48(1):6–16.
20. Tommelein E, Mehuys E, Petrovic M, Somers A, Colin P, Boussery K. Potentially inappropriate prescribing in community-dwelling older people across Europe: A systematic literature review. Eur J Clin Pharmacol. 2015 Dec 1;71(12):1415–27.

21. Wallace E, Mcdowell R, Bennett K, Fahey T, Smith SM, Kritchevsky S. Impact of potentially inappropriate prescribing on adverse drug events, health related quality of life and emergency hospital attendance in older people attending general practice: A prospective cohort study. J Gerontol A Biol Sci Med Sci. 2017;72(2):271–7.
22. Laroche ML, Charmes JP, Nouaille Y, Picard N, Merle L. Is inappropriate medication use a major cause of adverse drug reactions in the elderly? Br J Clin Pharmacol. 2007 Feb;63(2):177–86.
23. Hyttinen V, Jyrkkä J, Valtonen H. A systematic review of the impact of potentially inappropriate medication on health care utilization and costs among older adults. Med Care. 2016;54(10):950–64.
24. Reich O, Rosemann T, Rapold R, Blozik E, Senn O. Potentially inappropriate medication use in older patients in swiss managed care plans: Prevalence, determinants and association with hospitalization. PLoS One. 2014;9(8):23–5.
25. Fu A, Jiang J, Reeves JH, Fincham J, Liu G, Perri M. Potentially inappropriate medication use and healthcare expenditures in the US community-dwelling elderly. Med Care. 2007;45(5):472–6.
26. Hohl CM, Nosyk B, Kuramoto L, Zed PJ, Brubacher JR, Abu-Laban RB, et al. Outcomes of Emergency Department Patients Presenting With Adverse Drug Events. Ann Emerg Med. 2011 Sep;58(3):270-279.e4.
27. Mair, A; Fernandez-Llimos, F; Alonso, A; Harrison, C; Hurding, S; Kempen, T; Kinnear, M; Michael, N; McIntosh, J; Wilson M. The Simpathy Consortium. Polypharmacy Management by 2030: a patient safety challenge. SIMPATHY Consortium. Coimbra; 2017.
28. Beijer HJM, De Blaey CJ. Hospitalisations caused by adverse drug reactions (ADR): A meta-analysis of observational studies. Pharmacy World and Science. 2002;24(2):46–54.
29. Lavan AH, Gallagher P. Predicting risk of adverse drug reactions in older adults Amanda. Ther Adv Drug Saf. 2016;7(1):11–22.
30. Crosbie B, O'Callaghan ME, O'Flanagan S, Brennan D, Keane G, Behan W. A real-time measurement of general practice workload in the Republic of Ireland: A prospective study. British Journal of General Practice. 2020 Jul 1;70(696):E489–96.
31. Reeve E, Gnjidic D, Long J, Hilmer S, Decline C. A systematic review of the emerging definition of "deprescribing" with network analysis: implications for future research and clinical practice. 2015;
32. Reeve E, Thompson W, Farrell B. Deprescribing: A narrative review of the evidence and practical recommendations for recognizing opportunities and taking action. Eur J Intern Med. 2017;38:3–11.
33. Scott IA, Hilmer SN, Reeve E, Potter K, Couteur D le, Rigby D, et al. Reducing inappropriate polypharmacy: The process of deprescribing. JAMA Intern Med. 2015;175(5):827–34.
34. Iyer S, Naganathan V, McLachlan AJ, Le Couteur DG. Medication withdrawal trials in people aged 65 years and older: A systematic review. Drugs Aging. 2008;25(12):1021–31.
35. Page AT, Clifford RM, Potter K, Schwartz D, Etherton-Beer CD. The feasibility and effect of deprescribing in older adults on mortality and health: a systematic review and meta-analysis. Br J Clin Pharmacol. 2016;583–623.
36. Hansen CR, O'Mahony D, Kearney PM, Sahm LJ, Cullinan S, Huibers CJA, et al. Identification of behaviour change techniques in deprescribing interventions: a systematic review and meta-analysis. Br J Clin Pharmacol. 2018;84(12):2716–28.

37. Rankin A, Cadogan CA, Patterson SM, Kerse N, Cardwell CR, Bradley MC, et al. Interventions to improve the appropriate use of polypharmacy for older people. Cochrane Database of Systematic Reviews. 2018 Sep 3;9:CD008165.
38. Potter K, Page A, Clifford R, Etherton-Beer C. Deprescribing: A guide for medication reviews. Journal of Pharmacy Practice and Research. 2016;46(4):358–67.
39. Reeve E, Shakib S, Hendrix I, Roberts MS, Wiese MD, Reeve BPharm Hons E. Review of deprescribing processes and development of an evidence-based, patient-centred deprescribing process. Br J Clin Pharmacol. 2014;
40. Zechmann S, Trueb C, Valeri F, Streit S, Senn O, Neuner-Jehle S. Barriers and enablers for deprescribing among older, multimorbid patients with polypharmacy: An explorative study from Switzerland. BMC Fam Pract. 2019;20(1):1–10.
41. Sirois C, Ouellet N, Reeve E. Community-dwelling older people’s attitudes towards deprescribing in Canada. Research in Social and Administrative Pharmacy. 2017;13(4):864–70.
42. Schiøtz ML, Frølich A, Jensen AK, Reuther L, Perrild H, Petersen TS, et al. Polypharmacy and medication deprescribing: A survey among multimorbid older adults in Denmark. Pharmacol Res Perspect. 2018;(e00431).
43. Jia Hao L, Omar MS, Tohit N. Polypharmacy and Willingness to Deprescribe Among Elderly with Chronic Diseases. Int J Gerontol. 2018 Dec 1;12(4):340–3.
44. Reeve E, Wolff JL, Skehan M, Bayliss EA, Hilmer SN, Boyd CM. Assessment of Attitudes Toward Deprescribing in Older Medicare Beneficiaries in the United States. JAMA Intern Med. 2018 Dec 1;178(12):1673–80.
45. Sum G, Hone T, Atun R, Millett C, Suhrcke M, Mahal A, et al. Multimorbidity and out-of-pocket expenditure on medicines: A systematic review. Vol. 3, BMJ Global Health. BMJ Publishing Group; 2018. p. 505.
46. da Costa FA, Teixeira I, Duarte-Ramos F, Proença L, Pedro AR, Furtado C, et al. Effects of economic recession on elderly patients’ perceptions of access to health care and medicines in Portugal. Int J Clin Pharm. 2017 Feb 1;39(1):104–12.
47. Legido-Quigley H, Karanikolos M, Hernandez-Plaza S, de Freitas C, Bernardo L, Padilla B, et al. Effects of the financial crisis and Troika austerity measures on health and health care access in Portugal. Health Policy (New York). 2016;120(7):833–9.
48. Reeve E, Low LF, Hilmer SN. Beliefs and attitudes of older adults and carers about deprescribing of medications: a qualitative focus group study. The British journal of general practice. 2016 Aug 1;66(649):e552-60.
49. De Oliveira Martins S, Soares MA, Foppe Van Mil JW, Cabrita J. Inappropriate drug use by Portuguese elderly outpatients - Effect of the Beers criteria update. Pharmacy World and Science. 2006;28(5):296–301.
50. da Costa FA, Periquito C, Carneiro MC, Oliveira P, Fernandes AI, Cavaco-Silva P. Potentially inappropriate medications in a sample of Portuguese nursing home residents: Does the choice of screening tools matter? Int J Clin Pharm. 2016;38(5):1103–11.
51. Simões PA, Santiago LM, Maurício K, Simões JA. Prevalence of potentially inappropriate medication in the older adult population within primary care in Portugal: A nationwide cross-sectional study. Patient Prefer Adherence. 2019;13:1569–76.
52. Pérez-Jover V, Mira JJ, Carratala-Munuera C, Gil-Guillen VF, Basora J, López-Pineda A, et al. Inappropriate use of medication by elderly, polymedicated, or multipathological patients with chronic diseases. Vol. 15, International Journal of Environmental Research and Public Health. MDPI AG; 2018. p. 310.

53. JL W, KA S. Pharmacotherapy of gastric acidity, peptic ulcers, and gastroesophageal reflux disease. In: Brunton L, Chabner B, Knollman CB Editor Editor(s), editors. Goodman & Gilman's The Pharmacological Basis of Therapeutics. 12th ed. New York: McGraw-Hill; 2011. p. 1309–22.
54. Chan FK. Proton-pump inhibitors in peptic ulcer disease. Vol. 372, The Lancet. Elsevier Limited; 2008. p. 1198–200.
55. Kinoshita Y, Ishimura N, Ishihara S. Advantages and Disadvantages of Long-term Proton Pump Inhibitor Use. J Neurogastroenterol Motil. 2018;24(2):182.
56. Maes ML, Fixen DR, Linnebur SA. Adverse effects of proton-pump inhibitor use in older adults: a review of the evidence. Ther Adv Drug Saf. 2017;8(9):273.
57. Shimura S, Hamamoto N, Yoshino N, Kushiyama Y, Fujishiro H, Komazawa Y, et al. Diarrhea Caused by Proton Pump Inhibitor Administration: Comparisons Among Lansoprazole, Rabeprazole, and Omeprazole. Curr Ther Res Clin Exp. 2012 Jun;73(3):112–20.
58. Fohl AL. Proton pump inhibitor-associated pneumonia: Not a breath of fresh air after all? World J Gastrointest Pharmacol Ther. 2011 Jun 6;2(3):17.
59. Cunha N, Machado AP. Proton pump inhibitors and the risk of severe adverse events-A cardiovascular bombshell? Vol. 37, Revista Portuguesa de Cardiologia. 2018.
60. Forgacs I, Loganayagam A. Overprescribing proton pump inhibitors. Bmj. 2008;336(7634):1–2.
61. Bytzer P. Deprescribing proton pump inhibitors: why, when and how. Med J Aust [Internet]. 2018;209(10):436–8. Available from: https://doi.org/10.5694/mja18.00674
62. Hálfdánarson ÓÖ, Pottegård A, Björnsson ES, Lund SH, Ogmundsdottir MH, Steingrímsson E, et al. Proton-pump inhibitors among adults: a nationwide drug-utilization study. Therap Adv Gastroenterol. 2018 Jan 30;11:175628481877794.
63. All Wales Medicines Strategy Group, Safe Use of Proton Pump Inhibitors. February 2018.
64. Prescribing PPIs. Vol. 55, Drug and Therapeutics Bulletin. BMJ Publishing Group; 2017. p. 117–20.
65. Lee TJ, Fennerty MB, Howden CW. Systematic review: Is there excessive use of proton pump inhibitors in gastro-oesophageal reflux disease? Vol. 20, Alimentary Pharmacology and Therapeutics. 2004. p. 1241–51.
66. Dangler M, Ochs L, White R. Assessing the Appropriate Use of Proton Pump Inhibitors in a Veteran Outpatient Population. Federal Practitioner. 2013;30(5):21–5.
67. Alhawassi TM, Alatawi W, Alwhaibi M. Prevalence of potentially inappropriate medications use among older adults and risk factors using the 2015 American Geriatrics Society Beers criteria. BMC Geriatr. 2019 May 29;19(1).
68. Silva Rosália Oliveira A. Utilização de Inibidores da Bomba de Protões em Portugal. Análise ao período entre 2000 e 2016.
69. Infarmed. Monitorização do consumo de medicamentos. Meio Ambulatório. novembro de 2021. [Internet]. [cited 2022 Apr 22]. Available from: https://www.infarmed.pt/documents/15786/4373142/novembro/e120d720-d3a6-2b81-6be9-bcbcead0d946?version=1.0
70. Renom-Guiteras A, Meyer G, Thürmann PA. The EU(7)-PIM list: a list of potentially inappropriate medications for older people consented by experts from seven European countries.
71. Panel Expert By the American Geriatrics Society 2015 Beers Criteria Update. American Geriatrics Society 2019 Updated AGS Beers Criteria for Potentially Inappropriate Medication Use in Older Adults. J Am Geriatr Soc. 2019;00(21):1–21.

72. O'mahony D, O'sullivan D, Byrne S, O'connor MN, Ryan C, Gallagher P. STOPP/START criteria for potentially inappropriate prescribing in older people: version 2. Age Ageing. 2015;44:213–8.
73. Rodrigues DA, Herdeiro MT, Thürmann PA, Figueiras A, Coutinho P, Roque F. Operacionalização para Portugal da Lista EU(7)-PIM para Identificação de Medicamentos Potencialmente Inapropriados nos Idosos. Acta Med Port. 2020 Nov 23;33(13).
74. Lucchetti G, Lucchetti ALG. Inappropriate prescribing in older persons: A systematic review of medications available in different criteria. Arch Gerontol Geriatr. 2016;
75. Pruskowski JA, Springer S, Thorpe CT, Klein-Fedyshin M, Handler SM. Does Deprescribing Improve Quality of Life? A Systematic Review of the Literature. Drugs Aging. 2019;36(12):1097–110.
76. Pollock K, Grime J. Strategies for reducing the prescribing of proton pump inhibitors (PPIs): patient self-regulation of treatment may be an under-exploited resource. Soc Sci Med. 2000 Dec 15;51(12):1827–39.
77. Farrell B, Tsang C, Raman-Wilms L, Irving H, Conklin J, Pottie K. What Are Priorities for Deprescribing for Elderly Patients? Capturing the Voice of Practitioners: A Modified Delphi Process. Dalal K, editor. PLoS One. 2015 Apr 7;10(4):e0122246.
78. Zhang Y, Turner J, Martin P, Tannenbaum C. Does a Consumer-Targeted Deprescribing Intervention Compromise Patient-Healthcare Provider Trust? Pharmacy. 2018 Apr 16;6(2):31.
79. Martin P, Tamblyn R, Benedetti A, Ahmed S, Tannenbaum C. Effect of a Pharmacist-Led Educational Intervention on Inappropriate Medication Prescriptions in Older Adults The D-PRESCRIBE Randomized Clinical Trial. JAMA. 2018;320(18):1889–98.
80. Campins L, Serra-Prat M, Gózalo I, López D, Palomera E, Agustí C, et al. Randomized controlled trial of an intervention to improve drug appropriateness in communitydwelling polymedicated elderly people. Fam Pract. 2017;34(1):36–42.
81. Hanlon JT, Weinberger M, Samsa GP, Schmader KE, Uttech KM, Lewis IK, et al. A randomized, controlled trial of a clinical pharmacist intervention to improve inappropriate prescribing in elderly outpatients with polypharmacy. American Journal of Medicine. 1996 Apr 1;100(4):428–37.
82. Moriarty F, Cahir C, Bennett K, Fahey T. Economic impact of potentially inappropriate prescribing and related adverse events in older people: A cost-utility analysis using Markov models. BMJ Open. 2019;9(1):1–9.
83. Chau SH, Sluiter RL, Hugtenburg JG, Wensing M, Kievit W, Teichert M. Cost–Utility and Budget Impact Analysis for Stopping the Inappropriate Use of Proton Pump Inhibitors After Cessation of NSAID or Low-Dose Acetylsalicylic Acid Treatment. Drugs Aging. 2020;37(1):67–74.
84. Gillespie P, Clyne B, Raymakers A, Fahey T, Hughes CM, Smith SM. Reducing Potentially Inappropriate Prescribing for Older People in Primary Care: Cost-Effectiveness of the Opti-Script Intervention. Int J Technol Assess Health Care. 2017;33(4):494–503.
85. Zuidgeest MGP, Goetz I, Groenwold RHH, Irving E, van Thiel GJMW, Grobbee DE. Series: Pragmatic trials and real world evidence: Paper 1. Introduction. J Clin Epidemiol. 2017 Aug 1;88:7–13.
86. ARS | Norte. Observatório Regional de Saúde [Internet]. [cited 2022 Jul 4]. Available from: https://www.arsnorte.min-saude.pt/observatorio-regional-de-saude/perfis-de-saude/
87. Norma da Direção Geral de Saúde nº 036/2011. Supressão Ácida: Utilização dos Inibidores da Bomba de Protões e das suas Alternativas Terapêuticas. Direção-Geral da Saúde. Lisboa; 2011. p. 1–20.

88. Fajardo MA, Weir KR, Bonner C, Gnjidic D, Jansen J. Availability and readability of patient education materials for deprescribing: An environmental scan. Br J Clin Pharmacol. 2019;85(7):1396–406.
89. Masnoon N, Shakib S, Kalisch-Ellett L, Caughey GE. What is polypharmacy? A systematic review of definitions. BMC Geriatr. 2017;17(230):1–10.
90. EuroQol Group. EuroQol-a new facility for the measurement of health-related quality of life. Health Policy (New York). 1990 Dec 1;16(3):199–208.
91. Ferreira PL, Antunes P, Ferreira LN, Pereira LN, Ramos-Goñi JM. A hybrid modelling approach for eliciting health state preferences: the Portuguese EQ-5D-5L value set. Quality of Life Research. 2019;(0123456789).
92. Salgado T, Marques A, Geraldes L, Benrimoj S, Horne R, Fernandez-Llimos F. Cross-cultural adaptation of the Beliefs about Medicines Questionnaire into Portuguese. Sao Paulo Medical Journal. 2013;131(2):88–94.
93. Delgado AB, Lima ML. Contributo para a validação concorrente de uma medida de adesão aos tratamentos. Psicologia, Saúde & Doenças. 2001;2(2):81–100.
94. Glossary:Self-perceived health - Statistics Explained [Internet]. [cited 2020 Nov 19]. Available from: https://ec.europa.eu/eurostat/statistics-explained/index.php?title=Glossary:Self-perceived_health
95. Helgadottir H, Bjornsson ES. Problems Associated with Deprescribing of Proton Pump Inhibitors. Int J Mol Sci. 2019;20.
96. Graves T. Adverse Events After Discontinuing Medications in Elderly Outpatients. Arch Intern Med. 1997 Oct 27;157(19):2205.
97. Clyne B, Smith SM, Hughes CM, Boland F, Bradley M, Cooper JA, et al. Effectiveness of a Multifaceted Intervention for Potentially Inappropriate Prescribing in Older Patients in Primary Care: A Cluster-Randomized Controlled Trial (OPTI-SCRIPT Study). Ann Fam Med. 2015;13(6):545–53.
98. The Survey of Health, Ageing and Retirement in Europe (SHARE): Home [Internet]. [cited 2020 Nov 18]. Available from: http://www.share-project.org/home0.html
99. Akpan A, Roberts C, Bandeen-Roche K, Batty B, Bausewein C, Bell D, et al. Standard set of health outcome measures for older persons.
100. Montgomery DC. Introduction to Statistical Quality Control. 6th ed. John Wiley & Sons, INC; 2008.
101. RESPECT Trial Team RT, Bojke C, Philips Z, Sculpher M, Campion P, Chrystyn H, et al. Cost-effectiveness of shared pharmaceutical care for older patients: RESPECT trial findings. Br J Gen Pract. 2010 Jan;60(570):e20-7.
102. Drummond M, Sculpher M, Claxton K, Stoddart G, Torrance G. Methods for the economic evaluation of health care programme. Third edit. Oxford Medical Publications; 2005.
103. Devlin NJ, Brooks R. EQ-5D and the EuroQol Group: Past, Present and Future. Vol. 15, Applied Health Economics and Health Policy. Springer International Publishing; 2017. p. 127–37.
104. Ferreira LN, Ferreira PL, Pereira LN, Oppe M. The valuation of the EQ-5D in Portugal. Quality of Life Research. 2014 Mar 8;23(2)(2):413–23.
105. Torrance GW, Feeny D. Utilities and quality-adjusted life years. Int J Technol Assess Health Care. 1989;5(4):559–75.
106. Manca A, Hawkins N, Sculpher MJ. Estimating mean QALYs in trial-based cost-effectiveness analysis: The importance of controlling for baseline utility. Health Econ. 2005;14(5):487–96.

107. Stinnett AA, Mullahy J. Net Health Benefits : A New Framework for the Analysis of Cost-Effectiveness Analysis Uncertainty in. Medical Decision Making. 1998;18(2 Supplement):S68-80.
108. Fenwick E, Claxton K, Sculpher M. Representing uncertainty: The role of cost-effectiveness acceptability curves. Health Econ. 2001;10(8):779–87.

Annexes

Supplemental study materials – Stand-alone documents

Table 6 - List of stand-alone documents (Annexes)

Number	Document reference number	Date	Title
1	Annex 1	01/02/2021	Enrollment guide
2	Annex 2	15/12/2020	Informed Consent and leaflet (for IG and CG)
3	Annex 3	15/12/2020	Refusal form
4	Annex 4	01/02/2021	Baseline paper-based questionnaire
5	Annex 5	10/12/2020	Deprescription algorithm
6	Annex 6	22/06/2022	Educational booklet
7	Annex 7	10/12/2020	Therapeutic map
8	Annex 8	01/02/2021	Baseline telephone-based questionnaire
9	Annex 9	22/06/2022	Case report form – Patient passport
10	Annex 10	15/12/2020	Intervention log form (pharmacist)
11	Annex 11	22/06/2020	Intervention flowchart
12	Annex 12	15/12/2020	CRA monitoring form (digital)
13	Annex 13	01/02/2021	(3-month) telephone-based questionnaire
14	Annex 14	01/02/2021	(6-month) telephone-based questionnaire

The documents listed may be not the final versions. Minor adjustments to scripts and formatting procedures could be performed.
